# L-Rhamnose and Phenolic Esters-Based Monocatenar and Bolaform Amphiphiles: Eco-Compatible Synthesis and Determination of Their Antioxidant, Eliciting and Cytotoxic Properties

**DOI:** 10.3390/molecules28135154

**Published:** 2023-06-30

**Authors:** Emad Kordkatooli, Katia Bacha, Sandra Villaume, Stephan Dorey, Jean-Claude Monboisse, Sylvie Brassart-Pasco, Jean-Pierre Mbakidi, Sandrine Bouquillon

**Affiliations:** 1 Molecular Chemistry Reims Institute UMR CNRS 7312, Reims Champagne-Ardenne University, Boîte n° 44, B.P. 1039, 51687 Reims, France; emad.kordkatooli@etudiant.univ-reims.fr (E.K.); katia.bacha@etudiant.univ-reims.fr (K.B.); jean-pierre.mbakidi@univ-reims.fr (J.-P.M.); 2 RIBP—USC INRAE 1488, Université de Reims Champagne-Ardenne Reims, 51100 Reims, France; sandra.villaume@univ-reims.fr (S.V.); stephan.dorey@univ-reims.fr (S.D.); 3 Laboratoire de Biochimie Médicale et de Biologie Moléculaire, UMR CNRS/URCA 7369—Reims Champagne Ardenne University, UFR Médecine, 51 Rue Cognacq Jay, 51095 Reims, France; jean-claude.monboisse@univ-reims.fr (J.-C.M.); sylvie.pasco@univ-reims.fr (S.B.-P.)

**Keywords:** amphiphilic, monocatenar, bolaform, phenolic acids, rhamnosides, antioxidant, microwaves, plant immunity, dermal cytotoxicity

## Abstract

Symmetrical and dissymmetrical bolaforms were prepared with good to high yields from unsaturated L-rhamnosides and phenolic esters (ferulic, phloretic, coumaric, sinapic and caffeic) using two eco-compatible synthetic strategies involving glycosylation, enzymatic synthesis and cross-metathesis under microwave activation. The plant-eliciting activity of these new compounds was investigated in Arabidopsis model plants. We found that the monocatenar rhamnosides and bolaforms activate the plant immune system with a response depending on the carbon chain length and the nature of the hydrophilic heads. Their respective antioxidant activities were also evaluated, as well as their cytotoxic properties on dermal cells for cosmetic uses. We showed that phenolic ester-based compounds present good antioxidant activities and that their cytotoxicity is low. These properties are also dependent on the carbon chains used.

## 1. Introduction

**Figure 1:**
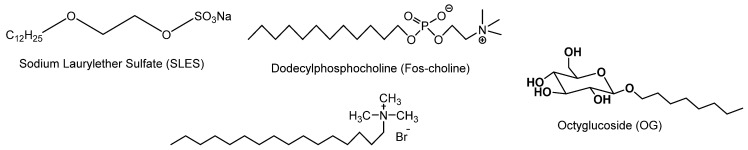
Examples of surfactants.

Surfactants are natural or synthetic amphiphilic compounds presenting specific polar and non-polar domains with typical solubility in water. They are able to reduce the interfacial tension of mixtures (oil and water) by adsorbing at the interfaces. Surfactants are classified into three large families according to the nature of their hydrophilic part [1]: ionic (cationic or anionic), zwitterionic (or amphoteric) and neutral (Figure 1) [2,3,4,5,6,7]. The malleability of their structure and the diversity of their properties allow them to be used in many products, particularly in household and industrial detergents, cosmetic formulations and as plant defense inducers [8,9,10,11]. Anionic surfactants (low molecular weight cation associated with sulphates, sulfonates or carboxylates) are the most widely used industrially, particularly in the field of detergents, thanks to their foaming properties [12,13,14,15,16]; cationic surfactants (trimethylated quaternary ammonium salts or pyridinium salts) [17,18,19] are active products in softeners (used to reduce static electricity) or shampoos.

Surfactants can also be classified into several categories according to their structure (number and arrangement of hydrophilic and hydrophobic poles within the molecule). The most common structure (in derivatives called monocatenar surfactants) displays a hydrophilic head and a hydrophobic chain. There are surfactants with several hydrophobic chains grafted onto the same hydrophilic head (double-stranded and three-stranded surfactants), but several hydrophilic heads can also be linked to one or more hydrophobic chains (called bolaform or twinned surfactants) [20].

In the current context of sustainable development, the lower availability of raw materials of petrochemical origin and consumers’ acceptance of a “bio-sourced surfactant” have increased interest in new surfactant technologies worldwide. Amphiphilic molecules from agro-resources are an innovative and interesting alternative for the substitution of petroleum-derived surfactants because they present relatively good biodegradability and low toxicity [21]. However, their production cost remains a limiting factor for their development [22]. Among the bio-based surfactants recently developed with these criteria, we can exemplify a sugar head containing alkyl polyglycosides (APG) [23,24,25].

Partially or completely bio-based surfactants are often anionic or non-ionic, including glycolipid, lipopeptide, phospholipid or even fatty acid types [26,27]. The hydrophilic head of the molecule can be a sugar, a carboxylic acid, an amino acid, an alcohol or peptides, while the lipophilic part is often a fatty acid or a fatty alcohol [28,29,30]. Bio-surfactants from agro-resource origins or plant bio-based surfactants are compounds that, in general, have one of the two hydrophilic or hydrophobic moieties of plant origin.

The synthesis of bio-sourced surfactants based on sugars has previously been described in the literature, particularly xylosides and rhamnosides [28,29,30,31,32,33,34]. Indeed, in 1993, 1,12-digluconamidododecane was synthetized from D-gluconolactone [35]. In 2004, Satgé and al. prepared a bolaform surfactant from D-galactose by a microwave-assisted glycosylation of a D-galactose protected by an unsaturated long-chain alcohol, followed by metathesis in the presence of the Grubbs I catalyst; hydrogenation in the presence of (Rh/Al_2_O_3_, H_2_) led to the saturated bolaform [36]. In 2010, K. Dzulkefly et al. synthesized symmetrical bolaform amphiphiles from an acid chloride with D-glucose as the hydrophilic head [37].

Sugar based-bolaamphiphiles have also been prepared, as exemplified by D-xyloside or L-rhamnosides-based bolaamphiphiles [31,32]. In a previous work [38], we described fatty ester-based bolaamphiphiles derived from phloretic acid and dissymmetric bolaamphiphiles with rhamnose and phloretic moieties as polar heads. The first step consisted of glycosylation of the sugar, followed by the enzymatic esterification of the phloretic acid with lipase B from Candida antarctica (CALB). Finally, a classical cross metathesis reaction using a Grubbs I catalyst led to the development of bolaform compounds. However, the kinetics of these reactions was low for an industrial application and the purification requires several chromatographies on silica columns, which are time-consuming and solvent-intensive processes.

**Figure 2:**
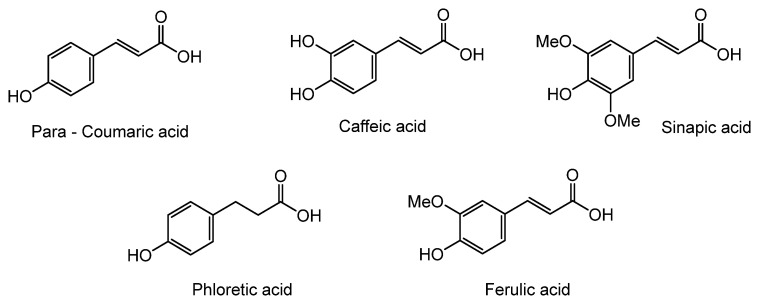
Structures of derivatives of hydroxycinnamic acids.

Herein, we discuss our newly developed approaches to obtain these bio-based compounds using two eco-compatible and fast synthetic methods coupling either glycosylation or enzymatic synthesis and activation by microwaves [39], which can reduce the reaction times and enhance the selectivity (few degradation products and appropriate quantities with no excess in one or the other substrates). Furthermore, greener solvents have been employed and demonstrated success as a reacting medium. For the processing and purification of the compounds, automated flash chromatography was used to considerably reduce the purification times. In these experiments, we compared old methodologies with the new approaches that we developed. Subsequently, we designed a panel of bio-based bolaforms from L-rhamnose (sugar from pectin) and other phenolic acid derivatives of lignin, such as phloretic, para-coumaric, ferulic, caffeic and sinapic acids, which could present better antioxidant properties (Figure 2).

The antioxidant properties of the monocatenar and bolaform adducts were then evaluated. Because rhamnose-based surfactants, classified as rhamnolipid mimetics, are well known for their antifungal properties and their ability to trigger an innate immune response in *Arabidopsis thaliana* [40], we also investigated the plant-eliciting properties of the monocatenar and bolaform adducts. Finally, cytotoxicity on dermal fibroblasts was also tested for a potential use in cosmetics.

## 2. Results and Discussion

### 2.1. Synthesis

**Scheme 1:**
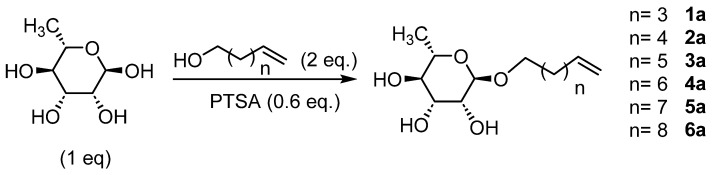
Glycosylation of L-rhamnose.

The glycosylation of L-rhamnose (Scheme 1) was initially performed with *p*-toluenesulfonic acid (PTSA) as a catalyst under conventional heating conditions with or without a solvent (Table 1). Low yields were obtained at 80 °C for 48 h (Table 1, entries 1 and 2). The time and temperatures were decreased to limit sugar degradation but similar results were obtained (Table 1, entries 3–6); however, the yields increased in the absence of a solvent, as the alcohol itself could be used as a solvent [2,3,4,5,6,7].

In order to increase the yield while avoiding the degradation of the starting reagents, we induced the reaction by microwaves [36,39,41]. First, the reaction conditions were optimized with a combination of L-rhamnose/Hex-5-enol using various conditions of time, temperature and power (Table 2).

The best results were obtained with an excess of four equivalents of 5-hexen-1-ol at a temperature of 60 °C for 2 h with a power of 60 W (Table 2, entry 8). The more homogeneous heating achieved by the microwaves improved the yield from 19% by conventional heating over 48 h to nearly 64% in only 2 h. It should also be noted that under these conditions, no degradation of rhamnose was observed. These same conditions were then used for the synthesis of L-rhamnosides of different chain lengths. The results obtained are summarized in Table 3.

**Scheme 2:**
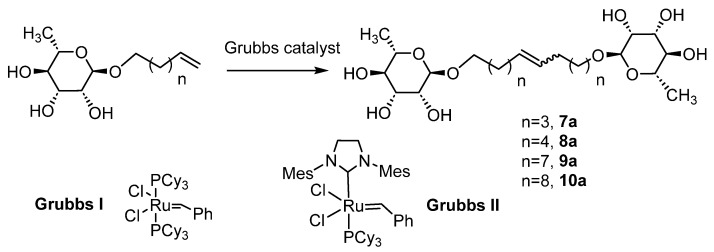
Metathesis of L-rhamnoside.

Rhamnosides were obtained with yields between 47 and 64%, which are inversely proportional to the chain length of the alkyl groups, as observed in the literature with sugars such as D-glucose, D-galactose and D-mannose [42]. The use of microwaves considerably reduces the reaction time and also improves the selectivity (few degradation products). Moreover, 2-methyltetrahydrofuran and γ-valerolactone were used as greener solvents [43] and they led to better yields, especially γ-valerolactone (Table 2, entries 2 and 8 vs. entries 1 and 7, respectively). This can be explained by the more polar and less viscous character of these solvents, which are favorable to the activation of the reagents under microwaves [44]. The formation of the rhamnosides was confirmed by IR (bands at 2926 cm^−1^ and 1640 cm^−1^, respectively, for the CH_3_ group of the rhamnose and the terminal alkene function on the unsaturated alcohols) and ^1^H NMR, with a shift at 1.25 ppm relative to the CH_3_ in the C6 position of rhamnose and signals between 4.52–4.72 ppm and 3.17–3.58 ppm for all the protons of rhamnose and at 4.98 ppm and 5.80 ppm for the terminal alkene function. The ^13^C NMR also confirmed the formation of rhamnosides with a terminal insaturation at 115.2 ppm and 139.2 ppm. In order to obtain bolaform compounds from these monocatenars, a cross-metathesis reaction (Scheme 2) was performed from rhamnosides using two types of Grubbs catalyst (Grubbs I and Grubbs II).

In the first step, we used classical conditions in the presence of Grubbs I with a conventional thermal activation. After several purification steps by silica chromatography, and with the addition of activated carbon and filtration of the celite, we obtained very low yields, lower than 20% (Table 4), regardless of the carbon chain length (Table 4, entries 1–3). These low yields could be explained not only by the purification steps or by the nature of the Grubbs I catalyst, which is less reactive compared to other generations, but also by the solvent used (the dichloromethane in which rhamnosides are not very soluble). The activation through microwaves also induced higher yields (37 and 52%, respectively, for carbon chains of 12 or 10 carbons; Table 4, entries 4 and 5) with a Z/E ratio of 20/80 as confirmed by NMR. These yields were again improved to 60 and 77% by using a Grubbs II catalyst and a mixture of CH_2_Cl_2_/MeOH (9/1) (Table 4, entries 7 and 8). Similar results have been also observed concerning the C18 chain (Table 4, entries 3, 6 and 9) and optimal conditions were used to prepare compound **10a** involving a C20 chain (Table 4, entry 10).

This synthetic methodology was extended for the preparation of bolaforms derived from hydroxycinnamic acids, which are widely used in cosmetics and are known for their antioxidant properties [45,46,47,48,49,50,51,52,53,54,55,56,57,58,59]. We used a method previously described in the literature [38] but improved the yields by using the CombiFlash technique for the purification. These bolaforms derived from cinnamic acids were prepared in two steps. The first step consists of synthesizing monocatenar esters from hydroxycinnamic acids and unsaturated fatty alcohols of different chain lengths. These monocatenar compounds were synthesized by a chemoenzymatic method in the presence of a lipase (lipase Novozym 435, lipase B from Candida antarctica “CAL-B”). According to the results of the literature [38], we chose 2-methyl-2-butanol as a solvent because of the stability and reactivity of the “CAL-B” enzyme. Polar alcohol allows for better chemical and thermal stability of the enzyme, but also avoids the possibility of having a competitive esterification with the starting phenolic acids.

Initial experiments with phloretic acid (used in excess amounts to obtain better yields) and alcohols of different unsaturated chain lengths (5-hexen-1-ol, 6-hepten-1-ol, 9-decen-1-ol 10-undecen-1-ol respectively) under the conditions described by Obounou et al. [38] showed low yields of phloretic acid esters despite good conversion (Table 5). Therefore, we modified the purification method by using a semiautomatic method with CombiFlash coupled with a UV detector at the output (UV1 = 280 nm and UV2 = 360 nm). After several trials, the elution program retained for all the products was as follows: a flow of 15 mL/min, 3 min eluting with 92% petroleum ether and 8% ethyl acetate, 27 min with 80% petroleum ether and 20% ethyl acetate and 32 min with 60% petroleum ether and 40% ethyl acetate. This eluting program allowed the recovery of the different pure monocatenars in a single step as opposed to a classical purification by flash chromatography, which requires several purification steps. This new purification method was very efficient in reducing product losses because the yields in the esters were closed to the conversions (Table 5).

After purification, these esters were characterized by infrared spectroscopy (IR), nuclear magnetic resonance (NMR) and mass spectrometry. In FTIR, we observed an elongation band at 1215 cm^−1^, characteristic of the phenol function; a band at 1640 cm^−1^, characteristic of the alkene function; and a band at 1704 cm^−1^ corresponding to the ester (C=O) function. The ^1^H NMR shows two doublets between 7.01 and 6.72 ppm (integrating for 8 H) corresponding to the aromatic protons, a multiplet for the protons of the alkene function between 5.82 and 4.95 ppm and a triplet at 4.02 ppm, which corresponds to the protons in alpha position of the ester function. The ^13^C NMR also confirms the presence of the aromatic ring (between 130 and 115 ppm), the carbonyl ring (173 ppm) and the alkene ring (131 ppm).

This methodology was extended to other phenolic acids (paracoumaric, ferulic, caffeic and sinapic). The first experiments carried out with these different acids in 2-methyl-2-butanol (2M2B) did not give any result for some of them (ferulic and sinapic acids) because of their very low solubility in 2M2B. Therefore, we used a mixture of 2M2B/THF (1/2) for caffeic acid and acetone for sinapic acid, respectively, as the solvents. However, the yields obtained from these acids were lower compared to those obtained with phloretic acid (Table 6). This result can be explained by the presence of an unsaturation in the α-position of the acid function, which reduces their reactivity towards enzymatic transformations [50,51]; this is not the case with phloretic acid.

In the second step, the synthesis of phloretic ester bolaforms was first performed by a metathesis in the presence of a Grubbs I catalyst added in 6 portions for 40 min at 45 °C and under microwave activation. These conditions led to low yields of C10 (20%), C12 (16%) and C18 (24%) bolaform compounds, respectively. We subsequently used the more reactive Grubbs II catalyst; after 40 min under microwave conditions, the yields were improved (Table 7). The same experiment was extended using the para-coumaric esters **5** and **6b**; good yields were obtained with a short carbon chain, while the yields remained relatively low with a longer chain (Table 7).

The compounds were classically characterized by IR, NMR, UV and elemental analysis. In IR, we identified the band at 1704 cm^−1^ of the carbonyl group, the band between 1640–1690 cm^−1^ corresponding to the double bond and the elongation band at 1215 cm^−1^, characteristic of the phenol function. In NMR, the singlet at 5.37 ppm integrating the two protons was also significant for the alkene function.

**Scheme 3:**
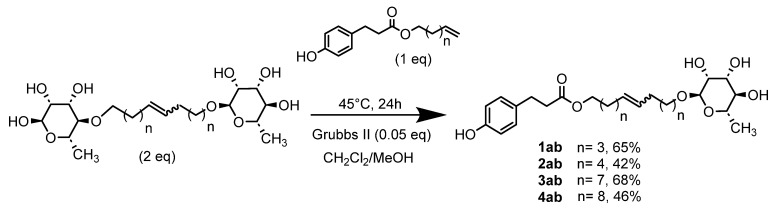
Cross-metathesis between rhamnosides and phenolic esters.

In order to obtain a system with both a good hydrophilic character (necessary, for example, for a good interaction with phospholipid membranes) and good antioxidant activity, we considered dissymmetric bolaform surfactants with a glycosylated head (L-rhamnose) and the other based on a hydroxycinnamic acid derivative. These dissymmetric bolaform surfactants were prepared by cross-metathesis. Different methods were tested according to the protocols described in the literature using different Ru-based catalysts, different ratios of rhamnosides/fatty acid esters or reaction conditions (time or temperature) [52]. Dissymmetric bolaamphiphiles were always obtained in a minority compared to the symmetric ones. However, inspired by the work of Blechert and Obounou et al. [38,53], four dissymmetric bolaamphiphiles were obtained under classical heating conditions and in the presence of Grubbs II (Scheme 3).

These dissymmetrical bolaforms were thus prepared by cross-metathesis of the symmetrical L-Rhamnose-based bolaform and the derived unsaturated phloretic esters. The use of a CH_2_Cl_2_/MeOH solvent mixture significantly improved the solubility of the reactants in contrast to CH_2_Cl_2_. TLC monitoring showed that the predominant compound formed was the dissymmetrical bolaform with a small amount of symmetrical bolaform derived from the unsaturated phloretic esters. After rapid purification (35 min) by automated flash chromatography, bolaforms **1ab**–**4ab** were obtained in yields up to 70% in 24 h.

These dissymmetric bolaforms were characterized by classical spectroscopic and spectrometric methods: IR (1615, 1732 and 3353 cm^−1^, respectively, for the C=C, C=O and OH bonds), NMR (5.80 ppm for the protons of the C=C, 2 doublets between 6.99 and 6.65 ppm for the aromatic protons and signals between 3.12 and 3.54 ppm for the hydrogens of the rhamnose, except the methyl group), UV and elemental analysis.

### 2.2. Antioxidant Properties

**Scheme 4:**
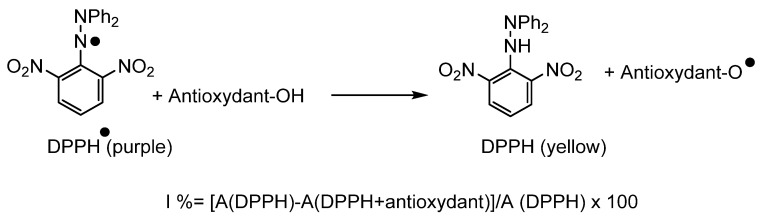
Reaction of an antioxidant with the DPPH● radical.

To evaluate the antioxidant activity, several methods [54,55] exist in the literature demonstrating the use of 2,2’-azinobis-3-ethylbenzothiazoline-6-sulfonic acid (ABTS) [56], β-carotene [57], Trolox (ORAC method) [58] and 2,2-diphenyl-1-picrylhydrazyl DPPH [59] (Scheme 4), respectively. The latter is based on the reduction of DPPH●, which is accompanied by a significant color change from purple to yellow, monitored by UV-visible spectroscopy at an absorption wavelength of 517 nm. We used this method to determine the antioxidant activity of our surfactants.

The antioxidant activity of phenolic ester and the derived bolaforms were evaluated in methanol by measuring their ability to reduce the DPPH● at a concentration of 10^−4^ mol·L^−1^. These activities were expressed as percent of inhibition according to the formula given in Scheme 4; vitamin C was used as a positive control. The antioxidant tests were performed at 45 min and in triplicate to confirm the repeatability of the results.

Preliminary observations showed that the monocatenar phloretic esters **1b** and **3b** have no antioxidant activity, but corresponding bolaforms **12b** and **14b** presented rather interesting antioxidant activity (Table 8, entries 8 and 9). In addition, the antioxidant activity of ferulic ester is greater than that of the phloretic ester containing the same carbon chain (Table 8, entries 5 and 7). This difference could be explained by the low stability of the phenoxy radical resulting from phloretic ester compared to the radical derived from ferulic ester, which could be more stabilized through the methoxy function in the ortho position. These results show that the antioxidant activity depends on both the stability of the resulting radical and the number of phenolic moieties.

In order to better understand the structure/antioxidant activity relationship of monocatenar esters, we have extended our measurements to para-coumaric, caffeic and sinapic esters; the values of IC50 are summarized in Table 8.

With the same carbon chain length, we observed that the monocatenar ester of caffeic acid presents the best antioxidant activity before the sinapic, the coumaric and the ferulic esters, respectively (Table 8, entries 2–5). We can therefore deduce that the number of OH groups on the aromatic ring has a great influence on the antioxidant power, as well as the double bond in the α position of the ester (compared to the phloretic ester, which shows no antioxidant activity). This ranking of antioxidant activities corresponds fairly well to one of the acids alone, except for the derivatives of sinapic acid because it has higher antioxidant activities alone than all the other acids [60]. The length of the carbon chain also seems to play an important role because we can note that the antioxidant powers are higher for a chain in C10 than for a chain in C6 (Table 8, entries 6 and 7 vs. entries 2 and 5), which could be explained by a more important inductive effect of a chain in C10, which stabilizes the radical.

Moreover, the antioxidant activity of dissymmetric bolaforms **1ab** and **3ab** was evaluated, but the study revealed no antioxidant activity, which is not very surprising considering that the phenolic part of the bolaform is constituted of phoretic acid. The solubility, on the other hand, is enhanced by the presence of the rhamnoside entity. Thus, it would be interesting to develop dissymmetrical bolaforms based on caffeic or sinapic esters and rhamnosides in a future study.

In conclusion, we have five compounds (**7b**, **6b**, **9b**, **11b** and **15b**) that present antioxidant activity superior to that of ascorbic acid (Table 8, entry 1). Furthermore, some of these compounds (**7b** and **15b**) have a very interesting hydrophilic character, which may demonstrate them as good candidates for cosmetic applications.

### 2.3. Plant-Eliciting Properties

As rhamnolipid- and xylolipid-based bolaforms have previously been characterized as plant defense inducers [40], we evaluated the eliciting properties of our new compounds in the plant model *Arabidopsis thaliana*. Rhamnolipids bolaforms were found to differentially activate early and late immunity-related plant defense responses, depending on the carbon chain length [40]. In this previous study, the 1′,14′-bis-tetradec-7′-enyl-L-rhamnopyranoside was proven to be the best candidate by combining the right chain length, an unsaturation and rhamnose as sugar.

Here, we aimed to compare the eliciting activities of monocatenar and bolaform rhamnosides (**1**–**10a**) to the dissymmetric bolaform **4ab** in order to observe a potential synergy between phenolic and rhamnose moieties. To investigate the immune response of compounds **1**–**10a** and **4ab** in *Arabidopsis thaliana*, we monitored the extracellular reactive oxygen species (ROS) production [40].

**Figure 3:**
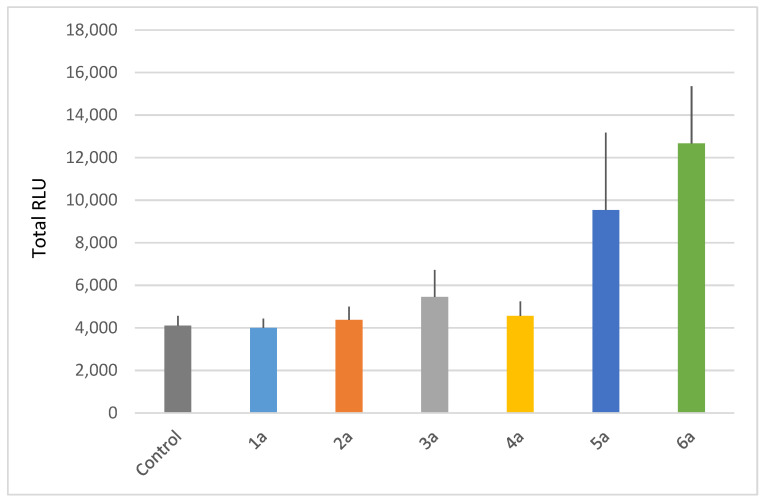
Plant-eliciting activities of monocatenar rhamnosides. Extracellular reactive oxygen species (ROS) production following treatment of Arabidopsis with C6 to C11 rhamnosides. Production of ROS was measured in leaf disks following treatment with the synthetic glycolipids at 100 µM. MeOH (0.5%) was used as a control. ROS production was measured using the chemiluminescence of luminol, and photon counts were expressed as relative luminescence units (RLUs). Histograms were calculated as the total RLUs over 12 h of monitoring. Data are mean (*n* = 6). Experiments were independently realized twice with similar results.

Our results show that only monocatenar rhamnosides with carbon chains containing 10 or 11 atoms are active on *Arabidopsis thaliana*, as compounds **5a** and **6a** triggered significant ROS production in planta (Figure 3).

**Figure 4:**
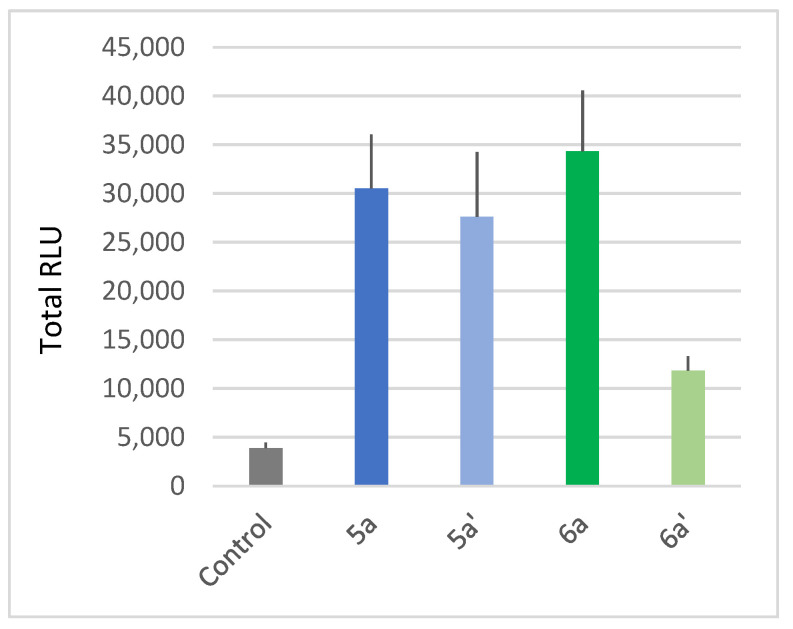
Plant-eliciting activities comparison between saturated and unsaturated monocatenar rhamnosides. Production of ROS was measured in leaf disks following treatment with the synthetic glycolipids at 100 µM. MeOH (0.5%) was used as a control. ROS production was measured using the chemiluminescence of luminol, and photon counts were expressed as relative luminescence units (RLUs). Histograms were calculated as the total RLUs over 12 h of monitoring. Data are mean ± SEM (*n* = 6). Experiments were independently realized twice with similar results.

We also compared the activity of the unsaturated and saturated compounds, respectively, **5a** and **6a** towards **5a′** and **6a′** obtained from **5a** and **6a** through classical Pd-catalyzed hydrogenation [61]. Our results show that the unsaturation of lipid chains leads to a stronger ROS response (Figure 4). Therefore, these data highlight the importance of combined long chains and unsaturation to enhance a monocatenar rhamnoside-triggered immune response in *Arabidopsis*.

**Figure 5:**
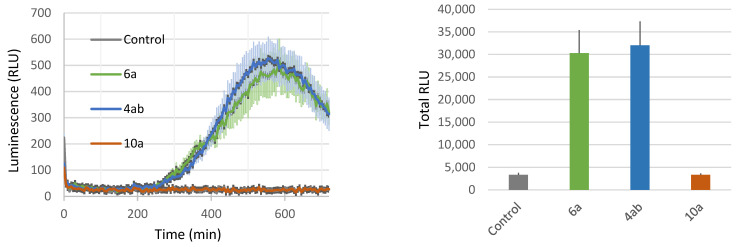
Plant-eliciting activities comparison between C11 rhamnoside, C20 dissymmetrical and symmetrical bolaforms. Production of ROS was measured in leaf disks following treatment with the synthetic glycolipids at 100 µM. MeOH (0.5%) was used as a control. ROS production was measured using the chemiluminescence of luminol and photon counts were expressed as relative luminescence units (RLUs). Kinetics of production (**left panel**) and sum of RLU from the same samples (**right panel**) are shown. Data are mean ± SEM (*n* = 6). Experiments were independently realized twice with similar results.

In addition, we found that the dissymmetrical C20 bolaform **4ab** induces ROS production close to the level measured for its monocatenar analogue **6a** (Figure 5). The symmetrical C20 bolaform **10a** was inactive at a concentration of 100 µM. In our previous study, we showed that symmetrical bolaform C18 (**9a**) was inactive and that the C10 bolaform (**7a**) was only slightly active at this concentration [40]. This robust immune response obtained with the dissymmetric bolaform may be explained by the presence of the phloretic acid, a relatively close analogue to salicylic acid, a well-known plant signal [62,63].

Altogether, our results demonstrate that monocatenar rhamnosides **5a** and **6a** and the dissymmetrical bolaform **4ab** can trigger a strong plant defense response in *Arabidopsis*, and therefore could be promising as plant-elicitor compounds.

### 2.4. Cytotoxicity Evaluation

As surfactants can be used in cosmetics, we completed our study with cytotoxicity tests on dermal fibroblasts. The toxicity of monocatenar and bolaform rhamnosides was evaluated on human dermal fibroblasts according to the protocol described in the experimental section. A fibroblast model is the best suited to the desired applications in cosmetics, specifically in the field of dermatology; we used the WST1 assay [64] to measure cell viability.

We first incubated dermal fibroblast cells for 48 h in the presence of our rhamnose-based compounds at increasing concentrations (1 to 1000 µg/mL). A dose-response presented by the residual dehydrogenase activity as a function of the surfactant concentration represented in mg/mL allowed us to determine the cytotoxic effect for each compound tested.

**Figure 6:**
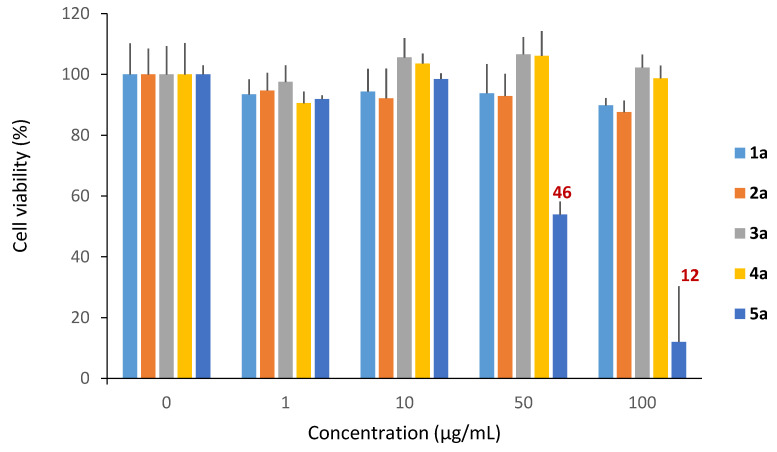
Cytotoxicity of monocatenar unsaturated rhamnosides.

In the concentration range tested, we observed a slight cytotoxicity of monocatenar rhamnosides **1**–**4a**; in contrast, **5a** (chain length of 10 carbons) showed a lower cell growth of about 46% starting from 50 µg/mL (Figure 6). This cytotoxicity could be due to the hydrophobic character of **5a**, which enables it to form micelles in the intracellular medium that can induce a disruption of the cell cycle and causing cell death by either apoptosis or necrosis [65].

This low toxicity of rhamnosides is in accordance with the use of rhamnose or rhamnose derivatives, which are known as promising biocompatible molecules for biomedical applications, e.g., in the internalization of nanoparticles with a therapeutic aim [66].

**Figure 7:**
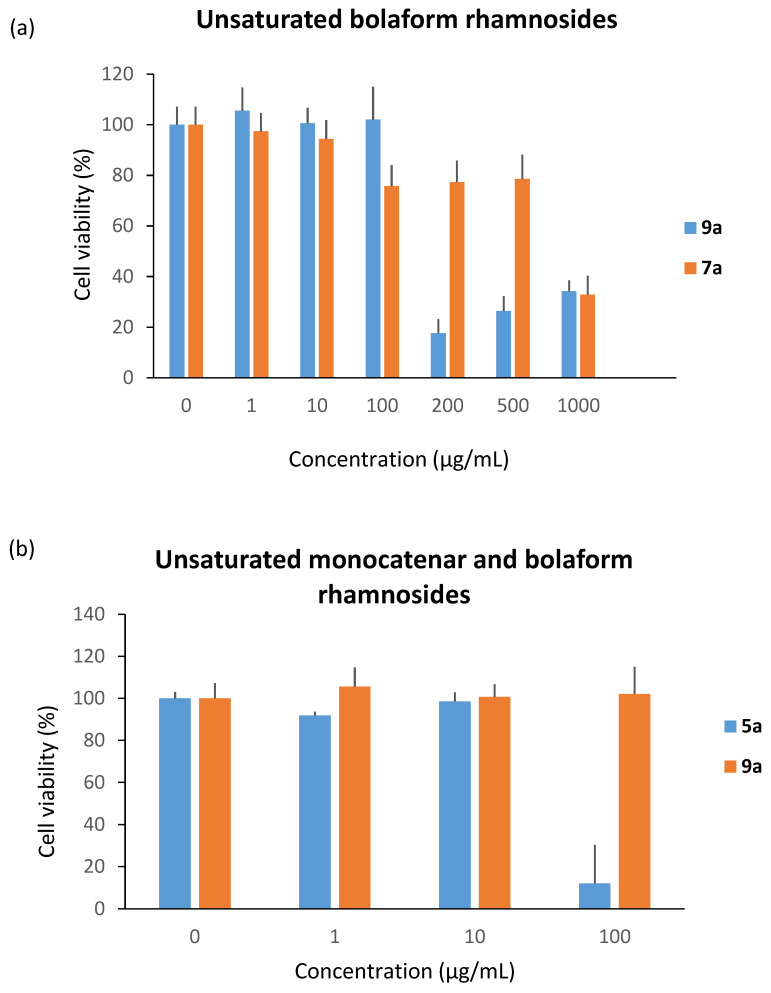
Cytotoxicity of (**a**) unsaturated bolaform rhamnosides and (**b**) monocatenar and bolaform rhamnosides.

We subsequently studied the cytotoxicity of the unsaturated bolaform rhamnosides **7a** and **9a** (Figure 7a). As discussed previously, the length of the carbon chain has a strong influence on the cytotoxic character; indeed, the results show that **9a** is five times more cytotoxic than **7a** (cytotoxicity at 200 µg/mL and 1000 µg/mL, respectively). Nevertheless, when we compared monocatenar rhamnosides and bolaform rhamnosides, i.e., **5a** and **9a**, the bolaamphiphilic structures clearly decrease the cytotoxicity compared to the monocatenar compounds (Figure 7b).

**Figure 8:**
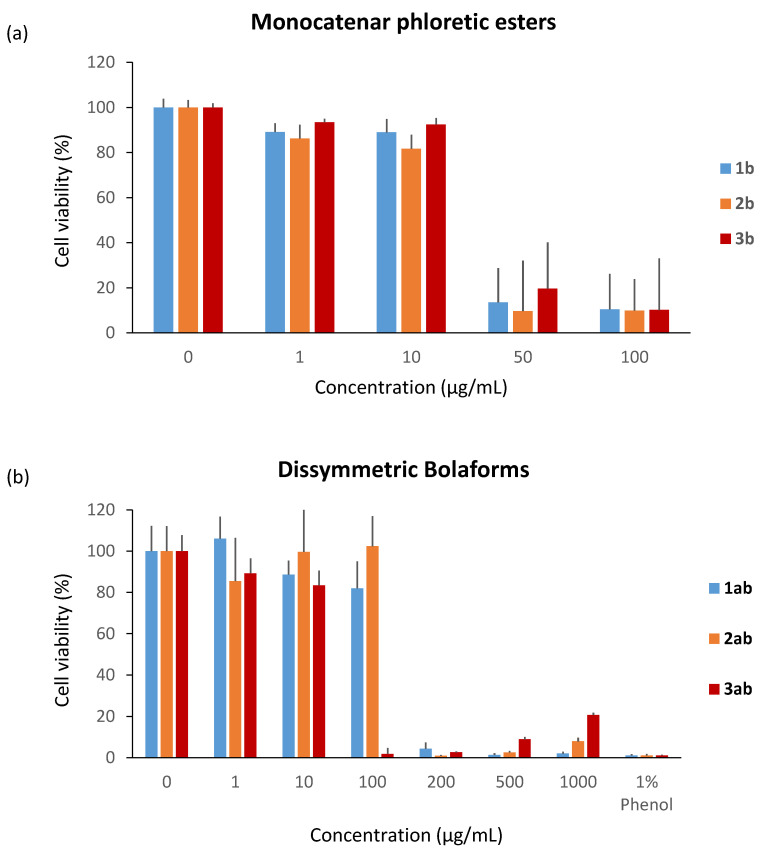
Cytotoxicity of (**a**) unsaturated monocatenar phloretic esters and (**b**) dissymmetric bolaforms.

Next, the cytotoxicity of unsaturated phloretic esters was evaluated under the same conditions at concentrations between 1 and 1000 µg/mL. In contrast to the monocatenar rhamnosides, all the unsaturated phloretic esters present cytotoxicity starting from 50 µg/mL (Figure 8a). This result can be explained by the intrinsic cytotoxicity of phloretic acid on specific cells, as previously described in the literature [67].

Regarding the boloform compounds, only the cytotoxicity of the dissymmetrical rhamnose/phloretic ester bolaforms could be evaluated because of the low solubility of the phloretic ester bolaforms in the culture medium. We also observed a decrease of the cytotoxicity when the phloretic esters are coupled with a monocatenar rhamnoside through a cross-metathesis step. In Figure 8b, we can observe low cytotoxicity for compounds **1ab** and **2ab** at 100 μg/mL, but high cytotoxicity for higher concentrations or for compound **3ab** at 100 μg/mL. This cytotoxicity decrease is most likely due to the presence of rhamnose, which is biocompatible with dermal cells and more hydrophilic, improving the solubility of the bolaform.

An in vitro study on dermal cells showed, first, a large difference concerning the cytotoxicity between the monocatenar compounds derived from L-rhamnose and those derived from phloretic acid and, secondly, the importance of the carbon chain length. Moreover, the bolaform structures (always depending on the carbon chain length) contribute to a reduction of the cytotoxic effect on dermal cells (even with bolaforms phloretic esters).

## 3. Experimental

All the reagents were commercially available and used as received. The solvents were dried and distilled under argon before use (CH_2_Cl_2_ over CaCl_2_ and THF over sodium/benzophenone) and stored over molecular sieves. The NMR spectra were recorded on a spectroscopic apparatus of the Bruker Avance Neo AC type (Wissembourg, France) (500 MHz for ^1^H, 126 MHz for ^13^C). The multiplicity of signals is cited according to s: singlet; sl: large singlet; d: doublet; t: triplet; q: quadruplet; m: multiplet; dt: doublet of triplets; dd: doublet of doublets; ddd: doublet of doublets of doublets; and td: triplet of doublets. The coupling constants (J) are expressed in Hertz (Hz). The chemical shifts (in ppm) for the ^1^H and ^13^C NMR spectra were referenced to residual protic solvent peaks. Elemental analyses (C, H and N) were carried out on a PerkinElmer 2400 C, H and N element analyzer. The IR spectra of the liquid and solid compounds were recorded on a Bruker Alpha-T FTIR (Wissembourg, France) spectrometer at room temperature. The ^1^H and ^13^C NMR spectra were recorded at room temperature with a Bruker AC 500 spectrometer (Wissembourg, France) (500 MHz for ^1^H, 62.5 MHz for ^13^C). The specific optical rotation ([α${]}_{D}^{20}$) of rhamnosides and rhamnoside-based boloamphiphiles or fatty ester and rhamnoside-based asymmetric bolaamphiphiles were measured using a polarimeter Anton Paar MCP 5100 (Les Ulis, France) at room temperature. Chromatography was carried out on SDS Silica 60 (40–63 nm), Art 2050044 (flash-chromatography) using the Reveleris^®^ X2 Flash Chromatography System from BUCHI. The microwave oven is a monomode CEM DISCOVERS-CLASS.

### 3.1. Cytotoxicity and Plant-Eliciting Activities

Normal human dermal fibroblasts were purchased from Promocell (Heidelberg, Germany). They were grown in DMEM supplemented with 10% fetal bovine serum (FBS), according to the manufacturer’s specifications, in Nunclon^®^ 75 cm^2^ flasks (Dutscher Brumath, France) at 37 °C in a humid atmosphere containing 5% CO_2_.

For the WST-1 assay, the cells were seeded in sterile 96-well microtiter plates (1 × 10,000 cells/well) and were allowed to settle for 24 h. Effectors were added to the cells at final concentrations ranging from 0 to 1000 µg/mL in DMEM supplemented with 0.5% FBS. The cells were incubated for 48 h and the medium was replaced by fresh medium containing 10% Wst-1 reagent. The absorbance was measured at 450 nm using a microplate spectrophotometer (SPECTROstar^®^ Nano, BMG Labtech, Champigny-sur-Marne, France) and the cell viability was calculated. The results are expressed as the mean ± standard deviation (*n* = 4). The plant-eliciting properties were monitored through the production of reactive oxygen species (ROS) in planta, as described in [22].

### 3.2. General Procedure for the Preparation of Rhamnosides under Microwave Activation with Tetrahydrofuran (THF) or 2-Methyltetrahydrofuran (2-MeTHF) as a Solvent

In a microwave tube, a solution of L-rhamnose and unsaturated alcohol in THF or 2-MeTHF (5 mL) was added at 60 °C with *p*-toluene sulfonic acid (PTSA). The mixture was stirred (600 rpm) under microwave irradiation for 2 h at a power of 60 W and a temperature of 60 °C. After 2 h of reaction, the reaction medium was neutralized with the addition of a 0.5 M MeONa solution (≈26 mL). After evaporation under reduced pressure, the purification of the rhamnosides was realized through a Reveleris^®^ X2 Flash Chromatography System by gradient elution of a CH_2_Cl_2_/MeOH mixture (8/2).

Hex-5′-enyl-α-L-rhamnopyranoside (**1a**)

The general procedure for the preparation of the rhamnosides under microwave activation with L-rhamnose (2 g; 10.35 mmol; 1 eq.), 5-Hexen-1-ol (5.27 mL; 41.4 mmol; 4 eq.) and PTSA (1.25 g; 6.21 mmol; 0.6 eq.) is as follows. Compound **1a** is obtained as a thick yellow liquid with a yield of 64% (with THF) and 63% (with 2-MeTHF).

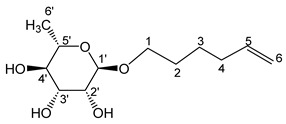



ν_max_ (ATR) cm^−1^: 3363 (OH), 2926–2856 (C–H), 1641 (C=C), 1384 (CH_3_), 1265–1231 (C–OH_Tert_.). [α${]}_{D}^{20}$ (589 nm, MeOH) = −52.501. ^1^H NMR (500 MHz; DMSO-d_6_, ppm) δ 1.25 (3H, d, *J* 7.5 Hz, **H6′**), 1.50 (2H, m, **H3**), 1.63 (2H, m, **H2**), 2.13 (2H, m, **H4**), 3.17–3.58 (5H, m, **H1**, **H2′**, **H3′**, **H4′**, **H5′**), 4.52–4.72 (3H, s, 3 O**H**), 4.98 (2H, d, *J* 7.2 Hz, **H6**), 5.80 (1H, m, **H5**). ^13^C NMR (125 MHz; DMSO-d_6_, ppm) δ 18.4 (**C6′**), 25.4 (**C3**), 29.1, 33.4 (**C2**, **C4**), 66.6 (**C1**), 68.9–73.9 (**C2′**, **C3′**, **C4′**, **C5′**), 100.6 (**C1′**), 115.3 (**C6**), 139.1 (**C5**). Analysis (%): calculated for: C_12_H_22_O_5_: C 58.52, H 9.02. Found: C 58.97, H 8.88.

Hept-6′-enyl-α-L-rhamnopyranoside (**2a**)

The general procedure for the preparation of the rhamnosides under microwave activation with L-rhamnose (0.76 g; 3.93 mmol; 1 eq.), 6-hepten-1-ol (3 mL; 15.72 mmol; 4 eq.) and PTSA (1.25 g; 6.21 mmol; 0.6 eq.) is as follows. Compound **2a** is obtained as a thick yellow liquid with a yield of 60%.

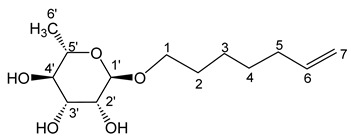



ν_max_ (ATR) cm^−1^: 3368 (OH), 2926–2856 (C–H), 1641 (C=C), 1382 (CH_3_),1227 (C–OH_Tert_.). [α${]}_{D}^{20}$ (589 nm, MeOH) = −49.101. ^1^H NMR (500 MHz; DMSO-d_6_, ppm) δ 1.13 (3H, d, *J* 7.5 Hz, **H6′**), 1.36 (4H, m, **H3**, **H4**), 1.50 (2H, m, **H2**), 2.02 (2H, m, **H5**), 3.17–3.56 (5H, m, **H1**, **H2′**, **H3′**, **H4′**, **H5′**), 4.52–4.73 (3H, s, 3 O**H**), 4.98 (2H, d, *J* 7.2 Hz, **H7**), 5.80 (1H, m, **H6**). ^13^C NMR (125 MHz; DMSO-d_6_, ppm) δ 18.38 (**C6′**), 25.7 (**C4**), 28.5, 29.3, 33.6 (**C3**, **C2**, **C5**), 66.8 (**C1**), 68.9–72.5 (**C2′**, **C3′**, **C4′**, **C5′**), 100.4 (**C1′**), 115.2 (**C7**), 139.2 (**C6**). Analysis (%): calculated for: C_13_H_24_O_5_: C 59.98, H 9.29. Found: C 59.71, H 9.14.

Oct-7′-enyl-α-L-rhamnopyranoside (**3a**)

The general procedure for the preparation of the rhamnosides under microwave activation with L-rhamnose (0.6 g; 3.11 mmol; 1 eq.), 7-octen-1-ol (2.52 mL; 12.4 mmol; 4 eq.) and PTSA (1.25 g; 6.21 mmol; 0.6 eq.) is as follows. Compound **3a** is obtained as a thick yellow liquid with a yield of 50%.

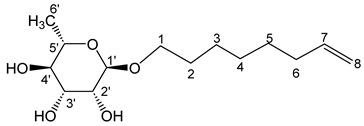



ν_max_ (ATR) cm^−1^: 3371 (OH), 2926–2856 (C–H), 1641 (C=C), 1381 (CH_3_),1231 (C–OH_Tert_.). [α${]}_{D}^{20}$ (589 nm, MeOH) = −46.501. ^1^H NMR (500 MHz; DMSO-d_6_, ppm) δ 1.13 (3H, d, J 7.5 Hz, **H6′**), 1.30 (6H, m, (**H4**, **H3**, **H5**), 2.02 (2H, m, **H2**), 3.18 (2H, m, **H6**), 3.36–3.58 (5H, m, **H1**, **H2′**, **H3′**, **H4′**, **H5′**), 4.53–5.02 (3H, s, 3 O**H**), 5.18 (2H, d, J 7.2 Hz, **H8**), 5.80 (1H, m, **H7**). ^13^C NMR (125 MHz; DMSO-d_6_) 18.4 (**C6′**), 26.0 (**C4**), 28.7, 28.8 29.5, 33.6 (**C3**, **C5**, **C2**, **C6**), 66.78 (**C1**), 68.9–72.5 (**C2′**, **C3′**, **C4′**, **C5′**), 100.4 (**C1**), 115.2 (**C8**), 139.3 (**C7**). Analysis (%): calculated for: C_14_H_26_O_5_: C 61.29, H 9.55. Found: C 60.83, H 9.41.

Non-8′-enyl-α-L-rhamnopyranoside (**4a**)

The general procedure for the preparation of the rhamnosides under microwave activation with L-rhamnose (0.57 g; 2.95 mmol; 1 eq.) and 8-nonen-1-ol (2.5 mL; 11.80 mmol; 4 eq.), and PTSA (1.25 g; 6.21 mmol; 0.6 eq.) is as follows. Compound **4a** is obtained as a thick yellow liquid with a yield of **53**%.

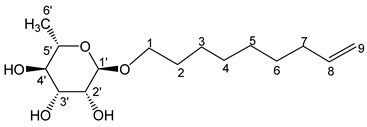



ν_max_ (ATR) cm^−1^: 3367 (OH), 2926–2856 (C–H), 1641 (C=C), 1382 (CH_3_),1230 (C–OH_Tert_.). [α${]}_{D}^{20}$ (589 nm, MeOH) = −45.001. ^1^H NMR (500, 1 MHz; CD_3_OD) δ 1.13 (3H, d, J 7.5 Hz, **H6′**), 1.32 (8H, m, **H5**, **H6**, **H4**, **H3**), 1.50 (2H, m, **H2**), 2.02 (2H, m, **H7**), 3.15–3.54 (5H, m, **H1**, **H2′**, **H3′**, **H4′**, **H5′**), 4.51–5.06 (3H, s, 3 O**H**), 5.27 (2H, d, J 7.2 Hz, **H9**), 5.80 (1H, m, **H8**). ^13^C NMR (125 MHz; DMSO-d_6_) δ 18.4 (**C6′**), 26.0 (**C5**), 26.1, 28.8, 29.1, 29.6, 33.6 (**C6**, **C4**, **C3**, **C2**, **C7**), 66.8 (**C1**), 68.8–73.2 (**C2′**, **C3′**, **C4′**, **C5′**), 100.5 (**C1**), 115.1 (**C9**), 139.3 (**C8**). Analysis (%): calculated for: C_15_H_28_O_5_: C 62.47, H 9.79. Found: C 62.35, H 9.65.

Dec-9′-enyl-α-L-rhamno-pyranoside (**5a**)

The general procedure for the preparation of the rhamnosides under microwave activation with L-rhamnose (0.53 g; 2.74 mmol; 1 eq.), 9-decen-1-ol (2.5 mL; 10.96 mmol; 4 eq.) and PTSA (1.25 g; 6.21 mmol; 0.6 eq.) is as follows. Compound **5a** is obtained as a thick yellow liquid with a yield of 48% (with THF) and 55% (with 2-MeTHF).

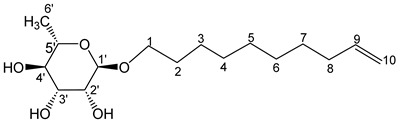



ν_max_ (ATR) cm^−1^: 3374 (OH), 2926–2856 (C–H), 1641 (C=C), 1382 (CH_3_),1229 (C–OH_Tert_.). [α${]}_{D}^{20}$ (589 nm, MeOH) = −43.701. ^1^H NMR (500, 1 MHz; CD_3_OD) δ 1.13 (3H, d, J 7.5 Hz, H6′), 1.32 (10H, m, H5, H6, H4, H7, H3), 1.50 (2H, m, H2), 2.02 (2H, m, H8), 3.15–3.54 (5H, m, H1, H2′, H3′, H4′, H5′), 4.15–4.54 (3H, s, 3 OH), 4.97 (2H, d, J 7.2 Hz, H10), 5.80 (1H, m, H9). ^13^C NMR (125 MHz; DMSO-d_6_) δ 18.4 (**C6′**), 26.1 (**C5**), 28.7, 28.9, 29.2, 29.3, 29.5, 33.6 (**C6**, **C4**, **C7**, **C3**, **C2**, **C8**), 66.8 (**C1**), 68.9–73.5 (**C2′**, **C3′**, **C4′**, **C5′**), 100.4 (**C1′**), 115.1 (**C10**), 139.3 (**C9**). Analysis (%): calculated for: C_16_H_30_O_5_: C 63.55, H 10.03. Found: C 63.16, H 9.82.

Undec-10′-enyl-α-L-rhamnopyranoside (**6a**)

The general procedure for the preparation of the rhamnosides under microwave activation with L-rhamnose (0.5 g; 2.59 mmol; 1 eq.), 10-undecen-1-ol (2.5 mL; 10.36 mmol; 4 eq.) and PTSA (1.25 g; 6.21 mmol; 0.6 eq.) is as follows. Compound **6a** is obtained as a thick yellow liquid with a yield of 47%.

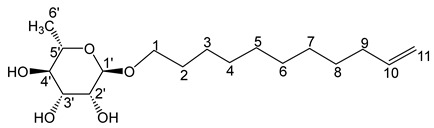



ν_max_ (ATR) cm^−1^: 3366 (OH), 2926–2856 (C–H), 1641 (C=C), 1382 (CH_3_),1229 (C–OH_Tert_.). [α${]}_{D}^{20}$ (589 nm, MeOH) = −37.001. ^1^H NMR (500, 1 MHz; CD_3_OD) δ 1.13 (3H, d, J 7.5 Hz, **H6′**), 1.31 (12H, **H6**, **H7**, **H5**, **H4**, **H8**, **H3**), 1.50 (2H, m, **H2**), 2.01 (2H, m,**H9**), 3.08–3.54 (5H, m, **H1**, **H2′**, **H3′**, **H4′**, **H5′**), 4.52–4.72 (3H, s, 3 O**H**), 4.97 (2H, d, J 7.2 Hz, **H11**), 5.80 (1H, m, **H10**). ^13^C NMR (125 MHz; DMSO-d_6_*)* δ 18.4 (**C6′**), 26.1 (**C6**), 28.7, 28.9, 29.2, 29.3, 29.4, 29.5, 33.6 (**C5**, **C7**, **C4**, **C8**, **C3**, **C2**, **C9**), 66.80 (**C1**), 68.9–72.5 (**C2′**, **C3′**, **C4′**, **C5′**),100.6 (**C1′**),115.1 (**C11**), 139.3 (**C10**). Analysis (%): calculated for C_17_H_32_O_5_: C 64.53, H 10.19. Found: C 63.91, H 10.14.

### 3.3. General Procedure for the Preparation of Rhamnosides under Microwave Activation with γ-Valerolactone as Solvent

The solution of L-rhamnose and unsaturated alcohol (5-Hexen-1-ol or 9-decen-1-ol) in γ-valerolactone was stirred at 60 °C with PTSA (*p*-toluenesulfonic acid) in a microwave tube. The mixture was irradiated under microwaves for 2 h at a power of 60 W and a temperature of 60 °C. After 2 h, the crude was extracted with diethylether (60 mL) after the addition of a NaCl-saturated solution (50 mL). The organic phase was dried over MgSO_4_ and evaporated under reduced pressure. The purification of the rhamnosides was then realized through the Reveleris^®^ X2 Flash Chromatography System by gradient elution of a CH_2_Cl_2_/MeOH mixture.

Compound **1a** was obtained as a thick yellow liquid with a yield of **66%** from L-rhamnose (2 g; 10.35 mmol; 1 eq.), 5-Hexen-1-ol (5.27 mL; 41.4 mmol; 4 eq.), 1.25 g of PTSA (6.21 mmol; 0.6 eq.) and γ-valerolactone (5 mL).

Compound **5a** was obtained as a thick yellow liquid with a yield of **73%** from L-rhamnose (1 g; 5.18 mmol; 1 eq.), 9-decen-1-ol (3.75 mL; 20.7 mmol; 4 eq.), 0.62 g of PTSA (3.11 mmol; 0.6 eq.) and γ-valerolactone (2.5 mL).

### 3.4. General Procedure for the Preparation of Rhamnoside-Based Boloamphiphiles under Microwave Activation

The rhamnoside was dissolved in a mixture of CH_2_Cl_2_/MeOH (8/2 mL) in a microwave tube under argon and the Grubbs II catalyst was added in three portions over the whole reaction time. The mixture was irradiated under microwaves for 40 min at a power of 60 W and a temperature of 60 °C. After 40 min of reaction, the reaction medium was treated with activated charcoal to remove the residual Grubbs catalyst or derivatives and was then filtered through celite. After evaporation, the residue was purified by the Reveleris^®^ X2 Flash Chromatography System with an elution mixture of CH_2_Cl_2_/MeOH (9/1) during 45 min.

1′,10′-bis-dec-5′-eny-L-rhamnopyranoside (**7a**)

The general procedure for the preparation of the rhamnoside-based bolaamphiphiles with compound **1a** (1.5 g, 6.09 mmol, 1 eq.) under microwave activation with Grubbs II catalyst (0.5 g, 0.6 mmol; 0.1 eq.) dissolved in 10 mL CH_2_Cl_2_/MeOH is as follows. Compound **7a** is obtained as a brown paste with a yield of **77%**.

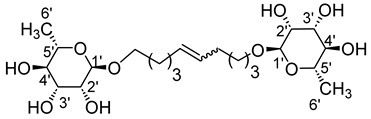



ν_max_ (ATR) cm^−1^: 3347 (OH), 2971–2901 (C–H), 1634 (C=C), 1384 (CH_3_),1128–1048 (C-O-C). [α${]}_{D}^{20}$ (589 nm, MeOH) = −34.801. ^1^H NMR (500 MHz; DMSO-d_6_, ppm) δ 1.24 (6H, d, J 7.5 Hz, **H6′**), 1.48 (4H, m, **H3**), 1.61 (4H, m, **H2**), 2.17(4H, m, **H4**), 3.56–3.12 (10H, m, **H1**, **H2′**, **H3′**, **H4′**, **H5′**), 4.70–4.48 (6H, s, 6 O**H**), 5.78 (2H, d, J 7.2 Hz, **H5**). ^13^C NMR (125 MHz; DMSO-d_6_, ppm) δ 18.3 (2 **C6′**), 23.6 (2 **C3**), 30.1, 33.2 (2 **C2**, 2 **C4**), 64.3 (2 **C1**), 67.6–71.9 (2 **C2′**, 2 **C3′**, 2 **C4′**, 2 **C5′**), 102.1 (2 **C1′**), 130.1 (CH_2_**CH**(**Z**)**=CH**(**Z**)CH_2_), 130.6 (CH_2_**CH**(**E**)**=CH**(**E**)CH_2_). Analysis (%): calculated for C_22_H_40_O_10_: C 56.88, H 8.68. Found: C 56.47, H 8.96.

1′,12′-bis-dodec-6′-enyl-l-rhamnopyranoside (**8a**)

The general procedure for the preparation of the rhamnoside-based bolaamphiphiles with compound **2a** (0.7 g, 2.69 mmol, 1 eq.) under microwave activation with Grubbs II catalyst (0.2 g, 0.27 mmol; 0.1 eq.) dissolved in 10 mL CH_2_Cl_2_/MeOH is as follows. Compound **8a** is obtained as a brown paste with a yield of 60%.

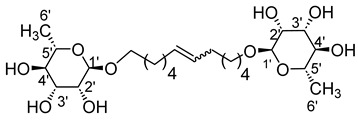



ν_max_ (ATR) cm^−1^: 3342 (OH), 2970–2913 (C–H), 1632 (C=C), 1386 (CH_3_), 1124–1051 (C–O–C). [α${]}_{D}^{20}$ (589 nm, MeOH) = −41.301. ^1^H NMR (500 MHz; DMSO-d_6_, ppm) δ 1.14 (6H, d, J 7.5 Hz, **H6′**), 1.38 (8H, m, **H3**, **H4**), 1.51 (4H, m, **H2**), 2.07 (4H, m, **H5**), 3.36–3.18 (10H, m, **H1**, **H2′**, **H3′**, **H4′**, **H5′**), 4.71–4.58 (6H, s, 6 O**H**), 5.78 (2H, d, J 7.2 Hz, **H5**). ^13^C NMR (125 MHz; DMSO-d_6_, ppm) δ 17.3 (2 **C6′**), 25.6 (2 **C3**), 29.0, 29.4, 33.6 (2 **C3**, 2 **C2**, 2 **C5**), 65.1 (2 **C1**), 68.6–72.6 (2 **C2′**, 2 **C3′**, 2 **C4′**, 2 **C5′**), 100.2 (2 **C1′**), 138.0 (CH_2_**CH**(**Z**)**=CH**(**Z**)CH_2_), 138.1 (CH_2_**CH**(**E**)**=CH**(**E**)CH_2_). Analysis (%): calculated for: C_24_H_44_O_10_: C 58.52, H 9.00. Found: C 58.87, H 9.19.

1′,18′-bis-octadec-9′-enyl-l-rhamnopyranoside (**9a**)

The general procedure for the preparation of the rhamnoside-based bolaamphiphiles with compound **5a** (1.45 g, 4.80 mmol, 1 eq.) under microwave activation with Grubbs II catalyst (0.4 g, 0.48 mmol; 0.1 eq.) dissolved in 10 mL CH_2_Cl_2_/MeOH is as follows. Compound **9a** is obtained as a brown paste with a yield of 56%.

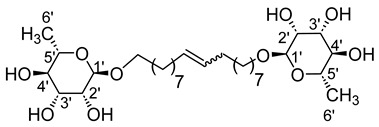



ν_max_ (ATR) cm^−1^: 3345 (OH), 2971–2922 (C–H), 1634 (C=C), 1382 (CH_3_), 1127–1051 (C–O–C). [α${]}_{D}^{20}$ (589 nm, MeOH) = −32.601. ^1^H NMR (500 MHz; DMSO-d_6_, ppm) δ 1.13 (6H, d, J 7.5 Hz, **H6′**), 1.34 (20H, m, **H3**, **H4**, **H5**, **H6**, **H7**), 1.51 (4H, m, **H2**), 2.09 (4H, m, **H8**), 3.16–3.58 (10H, m, **H1**, **H2′**, **H3′**, **H4′**, **H5′**), 4.31–4.58 (6H, s, 6 O**H**), 5.70 (2H, d, J 7.2 Hz, **H9**). ^13^C NMR (125 MHz; DMSO-d_6_, ppm) δ 18.3 (2 **C6′**), 26.2 (2 **C5**), 28.6, 28.9, 29.4, 29.7, 33.8 (2 **C3**, 2 **C2**, 2 **C4**, 2 **C5**, 2 **C6**, 2 **C7**, 2 **C8**), 66.8 (2 **C1**), 68.7–72.9 (2 **C2′**, 2 **C3′**, 2 **C4′**, 2 **C5′**), 100.4 (2 **C1′**), 130.1 (CH_2_**CH**(**Z**)**=CH**(**Z**)CH_2_), 130.5 (CH_2_**CH**(**E**)**=CH**(**E**)CH_2_). Analysis (%): calculated for C_30_H_56_O_10_: C 62.47, H 9.79. Found: C 62.11, H 9.29.

1′,20′-bis-eicosa-10′-enyl-l-rhamnopyranoside (**10a**)

The general procedure for the preparation of the rhamnoside-based bolaamphiphiles with compound **6a** (1.09 g, 3.60 mmol, 1 eq.) under microwave activation with Grubbs II catalyst (0.3 g, 0.36 mmol; 0.1 eq.) dissolved in 10 mL CH_2_Cl_2_/MeOH is as follows. Compound **10a** is obtained as a brown paste with a yield of 32%.

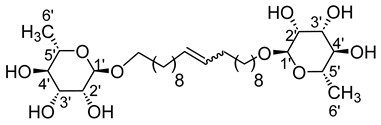



ν_max_ (ATR) cm^−1^: 3338 (OH), 2968–2919 (C–H), 1632 (C=C), 1381 (CH_3_), 1124–1053 (C–O–C). [α${]}_{D}^{20}$ (589 nm, MeOH) = −35.001. ^1^H NMR (500 MHz; DMSO-d_6_, ppm) δ 1.18 (6H, d, J 7.5 Hz, **H6′**), 1.39 (24H, m, **H3**, **H4**, **H5**, **H6**, **H7**, **H8**), 1.57 (4H, m, **H2**), 2.15 (4H, m, **H9**), 3.19–3.61 (10H, m, **H1**, **H2′**, **H3′**, **H4′**, **H5′**), 4.28–4.47 (6H, s, 6 O**H**), 5.74 (2H, d, J 7.2 Hz, **H10**). ^13^C NMR (125 MHz; DMSO-d_6_, ppm) δ 17.5 (2 **C6′**), 24.9 (2 **C5**), 27.4, 28.8, 29.1, 29.8, 32.5, 35.0 (2 **C3**, 2 **C2**, 2 **C4**, 2 **C5**, 2 **C6**, 2 **C7**, 2 **C8**, 2 **C9**), 66.5 (2 **C1**), 70.1–73.0 (2 **C2′**, 2 **C3′**, 2 **C4′**, 2 **C5′**), 107.3 (2 **C1′**), 131.3 (CH_2_**CH**(**Z**)**=CH**(**Z**)CH_2_), 133.1 (CH_2_**CH**(**E**)**=CH**(**E**)CH_2_). Analysis (%): calculated for C_32_H_60_O_10_: C 63.55, H 10.00. Found: C 63.41, H 9.89.

### 3.5. General Procedure for the Hydrogenation of Monocatenar Unsaturated Rhamnosides

The unsaturated rhamnoside (1 eq.) was dissolved in 4 mL of ethanol under an argon atmosphere. After 10 min of stirring, palladium on activated charcoal (Pd/C, 10% *w*/*w*, 0.02 eq.) was added and the solution was stirred for an additional 10 min under an argon atmosphere before being submitted to a H_2_ flow until completion (24 h at room temperature). Once the reaction was completed, the reaction mixture was filtered through celite. The obtained solution was then evaporated under reduced pressure. The saturated rhamnoside was obtained with quantitative yields.

Decyl-α-l-rhamno-pyranoside **(5a’)**

The general procedure for the hydrogenation of monocatenar unsaturated rhamnosides with unsaturated l-rhamnoside **5a** (0.4 g; 2.04 mmol; 1 eq.), palladium on activated charcoal (4.6 mg; 0.02 eq.) and 4 mL ethanol is as follows. Compound **5a′** is obtained as a thick yellow liquid with a quantitative yield.

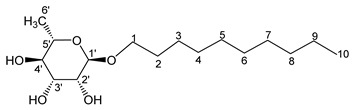



ν_max_ (ATR) cm^−1^: 3373 (OH), 2928–2854 (C–H), 1381 (CH_3_), 1227 (C–OH_Tert_). [α${]}_{D}^{20}$ (589 nm, MeOH) = −43.501. ^1^H NMR (500, 1 MHz; CD_3_OD) δ 0.86 (3H, t, J = 7.5 Hz, **H10**); 1.14 (3H, d, J 7.5 Hz, **H6′**), 1.32 (10H, m, **H3**, **H4**, **H5**, **H6**, **H7**, **H8**, **H9**), 1.51 (2H, m, **H2**), 3.14–3.56 (5H, m, **H1**, **H2′**, **H3′**, **H4′**, **H5′**), 4.12–4.57 (3H, s, 3 O**H**). ^13^C NMR (125 MHz; DMSO-d_6_) δ 14.1 (**C10**), 18.4 (**C6′**), 28.7, 28.9, 29.2, 29.3, 29.5, 33.6 (**C9**, **C8**, **C7**, **C6**, **C5**, **C4**, **C3**), 46.2 (**C2**), 66.8 (**C1**), 68.9–73.5 (**C2′**, **C3′**, **C4′**, **C5′**), 100.4 (**C1′**). Analysis (%): calculated for: C_16_H_32_O_5_: C 63.13, H 10.60. Found: C 63.32, H 10.72.

Undecyl-α-L-rhamnopyranoside (**6a′**)

The general procedure for the hydrogenation of monocatenar unsaturated rhamnosides with unsaturated l-rhamnoside **6a** (4 g; 2.34 mmol; 1 eq.), palladium on activated charcoal (4.4 mg; Pd/C 10% *w*/*w*; 0.02 eq.) is as follows. Compound **6a’** was obtained as a thick yellow liquid with a quantitative yield.

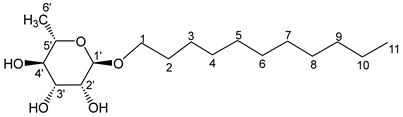



ν_max_ (ATR) cm^−1^: 3375 (OH), 2927–2854 (C–H), 1383 (CH_3_),1228 (C–OHTert). [α${]}_{D}^{20}$ (589 nm, MeOH) = −37.03. ^1^H NMR (500, 1 MHz; CD_3_OD) δ 0.85 (3H, t, J = 7.5 Hz, **H11**); 1.17 (3H, d, J 7.5 Hz, **H6′**), 1.34 (10H, m, **H3**, **H4**, **H5**, **H6**, **H7**, **H8**, **H9**, **H10**), 1.54 (2H, m, **H2**), 3.16–3.67 (5H, m, **H1**, **H2′**, **H3′**, **H4′**, **H5′**), 4.14–4.55 (3H, s, 3 O**H**). ^13^C NMR (125 MHz; DMSO-d_6_) δ 13.6 (**C11**), 18.3 (**C6′**), 27.5, 28.6, 28.9, 29.7, 30.5, 33.9 (**C10**, **C9**, **C8**, **C7**, **C6**, **C5**, **C4**, **C3**), 48.1 (**C2**), 67.1 (**C1**), 69.8–75.5 (**C2′**, **C3′**, **C4′**, **C5′**), 103.4 (**C1′**). Analysis (%): calculated for: C_17_H_34_O_5_: C 64.12, H 10.76. Found: C 64.25, H 10.64.

### 3.6. General Procedure for the Preparation of Monocatenar Esters Derived from Phenolic Acids

In a 250 mL flask, bicol was dissolved in phenolic acid (6 eq.) and unsaturated alcohol (1 eq.) in a solvent (2-methyl-2-butanol, or 2-methyl-2-butanol/THF or acetone, depending on the nature of the phenolic acid) under magnetic stirring. The resulting mixture was heated at 60 °C in the presence of 3 Ǻ molecular sieves (50 g·L^−1^). After adding an appropriate amount of the enzyme (2.5 g per 100 g of reaction mixture), the mixture was stirred for 48 h. Once the reaction was completed, the reaction mixture was filtered through celite to remove the enzyme; the resulting solution was evaporated under reduced pressure and the residue was purified on silica gel by the Reveleris^®^ X2 Flash Chromatography System with elution of a gradient petroleum ether/ethyl acetate.

Hex-5′-enyl-3-(4-hydroxyphenyl)propionic (**1b**)

The general procedure for the preparation of the monocatenar ester with 5-hexen-1-ol (0.7 mL; 6.53 mmol; 1 eq.) and 3-(4-hydroxyphenyl)propionic acid (6.50 g; 39.18 mmol; 6 eq.) in 60 mL of 2-methyl-2-butanol is as follows.

Compound **1b** was obtained as a clear oil with a yield of 95%.

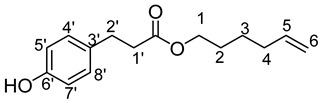



ν_max_ (ATR) cm^−1^: 3397 (OH), 2932 (C–H), 1732 (C=O), 1614 (C=C). ^1^H NMR (500 MHz; CD_3_OD, ppm) δ 1.36 (2H, broad, **H3**), 1.55 (2H, broad, **H4**), 2.03 (2H, broad, **H2**), 2.54 (2H, t, J 7.2 Hz, **H2′**), 2.77 (2H, t, J 7.2 Hz, **H1′**), 4.01 (2H, t, J 7.2 Hz, **H1**), 4.98 (2H, broad, **H6**), 5.78 (1H, broad, **H5**), 6.73 (2H, d, J 10 Hz, **H4′**, **H8′**), 7.00 (2H, d, J 10 Hz, **H5′**, **H7′**). ^13^C NMR (125 MHz; DMSO-d_6_, ppm) δ 25.0 (**C3**), 28.1 (**C4**), 30.0 (**C2**), 33.2 (**C2′**), 36.0 (**C1′**), 64.0 (**C1**),115.4 (**C4′**, **C8′**), 115.5 (**C5′**, **C7′**), 129.5 (**C6**), 131.0 (**C5**), 138.9 (**C3′**), 156.1 (**C6′**), 172.8 (**C=O**). Analysis (%): calculated for C_15_H_20_O_3_: C 72.55, H 8.12. Found: C 72.93, H 8.46.

Hept-6′-enyl-3-(4-hydroxyphenyl)propionic (**2b**)

The general procedure for the preparation of the monocatenar ester with 5-hepten-1-ol (0.47 mL; 3.01 mmol; 1 eq.) and 3-(4-hydroxyphenyl)propionic acid (3 g; 18.05 mmol; 6 eq.) in 60 mL of 2-methyl-2-butanol is as follows.

Compound **2b** was obtained as a clear oil with a yield of 88%.

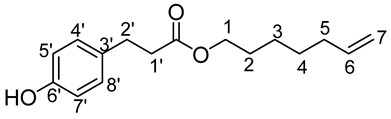



ν_max_ (ATR) cm^−1^: 3392 (OH), 2930 (C–H), 1732 (C=O), 1614 (C=C). ^1^H NMR (500 MHz; CD_3_OD, ppm) δ 1.29 (2H, m, **H4**), 1.38 (2H, m, **H3**), 1.56 (2H, m, **H5**), 2.03 (2H, m, **H2**), 2.55 (2H, t, J 7.2 Hz, **H2′**), 2.74 (2H, t, J 7.2 Hz, **H1′**), 3.98 (2H, t, J 7.2 Hz, **H1**), 5.03 (2H, m, **H7**), 5.83 (1H, m, **H6**), 6.66 (2H, d, J 10 Hz, **H5′**, **H7′**), 7.02 (2H, d, J 10 Hz, **H4′**, **H8′**). ^13^C NMR (125 MHz; DMSO-d_6_, ppm) δ 25.3 (**C4**), 28.3 (**C3**), 28.4 (**C5**), 30.0 (**C2**), 33.5 (**C2′**), 36.0 (**C1′**), 64.1 (**C1**),115.2 (**C7**), 115.5 (**C4′**, **C8′**), 129.5 (**C5′**, **C7′**), 131.0 (**C3′**), 139.1 (**C6**), 156.1 (**C6′**), 172.8 (**C=O**). Analysis (%): calculated for C_16_ H_22_O_3_: C 73.25, H 8.45. Found: C 72.93, H 8.24.

Dec-9′-enyl-3-(4-hydroxyphenyl)propionic (**3b**)

The general procedure for the preparation of the monocatenar ester with 9-decen-1-ol (1.2 mL; 3.01 mmol; 1 eq.) and 3-(4-hydroxyphenyl)propionic acid (6,51 g; 18.06 mmol; 6 eq.) in 60 mL of 2-methyl-2-butanol is as follows.

Compound **3b** was obtained as a clear oil with a yield of 96%.

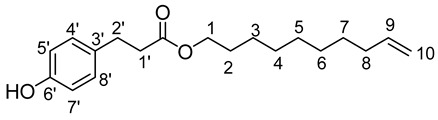



ν_max_ (ATR) cm^−1^: 3397 (OH), 2925 (C–H), 1733 (C=O), 1614 (C=C). ^1^H NMR (500 MHz; CD_3_OD, ppm) δ 1.26 (8H, m, **H4**, **H5**, **H6**, **H7**), 1.36 (2H, m, **H3**), 1.55 (2H, m, **H8**), 2.04 (2H, m, **H2**), 2.54 (2H, t, J 7.2 Hz, **H2′**), 2.77 (2H, t, J 7.2 Hz, **H1′**), 4.00 (2H, t, J 7.2 Hz, **H1**), 5.00 (2H, m, **H10**), 5.83 (1H, m, **H9**), 6.71 (2H, d, J 10 Hz, **H5′**, **H7′**), 6.99 (2H, d, J 10 Hz, **H4′**, **H8′**). ^13^C NMR (125 MHz; DMSO-d_6_, ppm) δ 25.8 (**C5**), 28.6 (**C6**), 28.8 (**C4**), 28.9 (**C7**), 29.1 (**C3**), 29.2 (**C8**), 30.1 (**C2**), 33.7 (**C2′**), 36.1 (**C1′**), 64.2 (**C1**), 115.0 (**C10**), 115.5 (**C4′**, **C8′**), 129.5 (**C5′**, **C7′**), 130.9 (**C3′**), 139.2 (**C9**), 156.1 (**C6′**), 172.7 (**C=O**). Analysis (%): calculated for C_19_ H_28_O_3_: C 74.96, H 9.27. Found: C 75.13, H 9.03.

Undec-10′-enyl-3-(4-hydroxyphenyl)propionic (**4b**)

The general procedure for the preparation of the fatty esters with 36.83 mmol of 3-(4-hydroxyphenyl)propionic acid (6.12 g; 6 eq.) and 6.14 mmol of 10-undecen-1-ol (1.045 g; 1 eq.) is as follow. Compound **4b** was obtained as a bright oil with a yield of 79%.

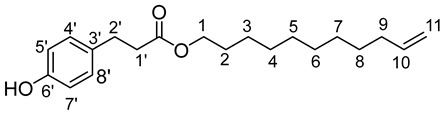



ν_max_ (ATR) cm^−1^: 3387 (OH), 2931 (C–H), 1732 (C=O), 1613 (C=C). ^1^H NMR (500 MHz; CD_3_OD, ppm) δ 1.24 (8H, m, **H4**, **H5**, **H6**, **H7**, **H8**), 1.34 (2H, m, **H3**), 1.58 (2H, m, **H9**), 2.07 (2H, m, **H2**), 2.51 (2H, t, J 7.2 Hz, **H2′**), 2.74 (2H, t, J 7.2 Hz, **C1′**), 3.97 (2H, t, J 7.2 Hz, **H1**), 5.21 (2H, m, **C11**), 5.81 (1H, m, **H10**), 6.65 (2H, d, J 10 Hz, **H5′**, **H7′**), 7.09 (2H, d, J 10 Hz, **H4′**, **H8′**). ^13^C NMR (125 MHz; DMSO-d_6_, ppm) δ 24.8 (**C5**), 28.1 (**C6**), 28.5 (**C4**), 28.8 (**C7**), 29.1 (**C3**), 29.2 (**C8**), 30.0 (**C9**), 30.1 (**C2**), 33.5 (**C2′**), 35.3 (**C1′**), 65.1 (**C1**), 114.0 (**C11**), 115.6 (**C4′**, **C8′**), 130.0 (**C5′**, **C7′**), 130.7 (**C3′**), 140.0 (**C10**), 154.6 (**C6′**), 172.1 (**C=O**). Analysis (%): calculated for C_20_ H_30_O_3_: C 75.43, H 9.50. Found: C 75.37, H 9.49.

Hex-5′-enyl-3-(4-hydroxyphenyl)prop-2-enoic (**5b**)

The general procedure for the preparation of the monocatenar ester with 5-hexen-1-ol (0.36 mL; 3.04 mmol; 1 eq.) and p-coumaric acid (3.28 g; 18.21 mmol; 6 eq.) in 40 mL of 2-methyl-2-butanol is as follows. Compound **5b** was obtained as a pale yellow oil with a yield of 70%.

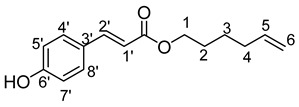



ν_max_ (ATR) cm^−1^: 3397 (OH), 2932 (C–H), 1732 (C=O), 1614 (–CH=CH_2_–), 1668 (Phenyl-**CH=CH**-COO). ^1^H NMR (500 MHz; CD_3_OD, ppm) δ 1.51 (2H, m, **H3**), 1.75 (2H, m, **H4**), 2.15 (2H, m, **H2**), 4.25 (2H, t, J 7.2 Hz, **H1**), 5.01 (2H, m, **H6**), 5.80 (1H, m, **H5**), 6.35 (1H, d, J 7.2 Hz, **H1′**), 6.80 (2H, d, J 7.2 Hz, **H7′**, **H5′**), 7.40 (1H, d, J 7.2 Hz, **H1′**), 7.65 (2H, d, J 7.2 Hz, **H4′**, **H8′**). ^13^C NMR (125 MHz; DMSO-d_6_, ppm) δ 23.2 (**C3**), 25.2 (**C4**), 34.2 (**C2**), 68.3 (**C1**), 114.2 (**C6**), 115.0 (**C1′**), 127.3 (**C5**), 130.1 (**C7′**, **C5′**), 140.1 (**C8′**, **C4′**), 145.0 (**C3′**), 158.0 (**C6′**), 168.4 (**C=O**). Analysis (%): calculated for C_15_H_18_O_3_: C 73.15, H 7.37. Found: C 72.77, H 7.66.

Dec-9′-enyl-3-(4-hydroxyphenyl)prop-2-enoïc (**6b**)

The general procedure for the preparation of the monocatenar ester with 9-decen-1-ol (0.57 mL; 3.2 mmol; 1 eq.) and para-coumaric acid (3.15 g; 19.2 mmol; 6 eq.) in 40 mL of 2-methyl-2-butanol is as follows. Compound **6b** was obtained as a yellow oil with a yield of 74%.

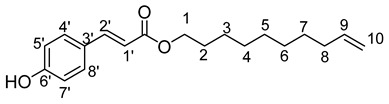



ν_max_ (ATR) cm^−1^: 3397 (OH), 2932 (C–H), 1732 (C=O), 1612 (–CH=CH_2_–), 1665 (Phenyl-**CH=CH**-COO). ^1^H NMR (500 MHz; CD_3_OD, ppm) δ 11.22 (8H, m, **H4**, **H5**, **H6**, **H7**), 1.70 (2H, m, **H3**), 2.10 (2H, m, **H8**), 3.75 (2H, m, **H2**), 4.20 (2H, t, J 7.2 Hz, **H1**), 5.01 (2H, d, J 7.2 Hz, **H10**), 5.81 (1H, m, **H9**), 6.32 (1H, d, J 7.2 Hz, **H1′**), 6.80 (2H, d, J 10 Hz, **H7′**, **H5′**), 7.43 (1H, d, J 7.2 Hz, **H2′**), 7.65 (2H, d, J 10 Hz, **H4′**, **H8′**). ^13^C NMR (125 MHz; DMSO-d_6_, ppm) δ 25.1 (**C5**), 27.5 (**C6**), 28.4 (**C4**), 28.9 (**C7**), 29.1 (**C3**), 29.5 (**C8**), 34.0 (**C2**), 77.2 (**C1**), 114.1 (**C10**), 115.2 (**C1′**), 116.0 (**C9**), 127.1 (**C5′**, **C7′**), 130.3 (**C4′**, **C8′**), 140.2 (**C9**), 145.1 (**C2′**), 158.7 (**C6′**), 168.9 (**C=O**). Analysis (%): calculated for C_19_ H_26_O_3_: C 75.46, H 8.67. Found: C 75.67, H 9.21.

Hex-5′-enyl-3-(3,5-dihydroxyphenyl)prop-2-enoic (**7b**)

The general procedure for the preparation of the monocatenar ester with 5-hexen-1-ol (0.36 mL; 3.04 mmol; 1 eq.) and caffeic acid (3.28 g; 18.24 mmol; 6 eq.) in 30 mL of 2-methyl-2-butanol/THF (10/20) is as follows. Compound **7b** was obtained as a pale yellow oil with a yield of 52%.

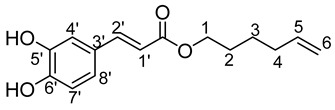



ν_max_ (ATR) cm^−1^: 3390 (OH), 2930 (C–H), 1734 (C=O), 1615 (–CH=CH_2_–), 1664 (Phenyl-CH=CH-COO). ^1^H NMR (500 MHz; CD_3_OD, ppm) δ 1.59 (2H, m, **H3**), 1.61 (2H, m, **H2**), 2.21 (2H, m, **H4**), 4.22 (2H, t, J 7.2 Hz, **H1**), 5.05 (2H, d, J 7.2 Hz, **H6**), 5.12 (1H, d, J 7.2 Hz, **H1′**), 5.85 (1H, m, **H5**), 6.36 (2H, d, J 7.2 Hz, **H2′**), 6.79 (1H, d, J 7.2 Hz, **H8′**), 6.94 (1H, d, J 7.2 Hz, **H7′**), 7.18 (1H, s, **C4′**), 7.50 (1H, d, J 7.2 Hz, **C2′**). ^13^C NMR (125 MHz; DMSO-d_6_, ppm) δ 24.9 (**C3**), 25.4 (**C4**), 33.9 (**C2**), 65.1 (**C1**), 115.2 (**C6**), 116.4 (**C1′**), 117.1 (**C7′**), 122.2 (**C8′**), 128.1 (**C3′**), 139.0 (**C5**), 144.2 (**C4′**), 147.2 (**C5′**, **C6′**), 167.4 (**C=O**). Analysis (%): calculated for C_15_H_18_O_4_: C 68.69, H 6.92. Found: C 68.24, H 7.98.

Dec-9′-enyl-3-(4-hydroxyphenyl)prop-2-enoic (**8b**)

The general procedure for the preparation of the monocatenar ester with 9-decen-1-ol (0.74 mL; 3.7 mmol; 1 eq.) and caffeic acid (4 g; 19.20 mmol; 6 eq.) in 30 mL of 2-methyl-2-butanol/THF (10/20) is as follows. Compound **8b** was obtained as a pale yellow oil with a yield of 45%.

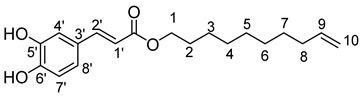



ν_max_ (ATR) cm^−1^: 3375(OH), 2926 (C–H), 1731 (C=O), 1614 (–CH=CH_2_–), 1661 (Phenyl-CH=CH–COO). ^1^H NMR (500 MHz; CD_3_OD, ppm) δ 1.28 (8H, m, **H4**, **H5**, **H6**, **H7**), 1.44 (2H, m, **H3**), 1.62 (2H, m, **H2**), 2.18 (2H, m, **H8**), 3.99 (2H, t, J 7.2 Hz, **H1**), 5.02 (2H, d, J 7.2 Hz, **H10**), 5.17 (1H, d, J 7.2 Hz, **H1′**), 5.83 (1H, m, **H9**), 6.30 (1H, d, J 10 Hz, **H8′**), 6.79 (1H, d, J 7.2 Hz, **H7′**), 6.95 (1H, d, J 7.2 Hz, **H4′**), 7.49 (1H, d, J 7.2 Hz, **H2′**). ^13^C NMR (125 MHz; DMSO-d_6_, ppm) δ 25.52 (**C5**), 29.32, 29.57, 31.16, 32.48 (**C6**, **C4**, **C7**, **C3**), 32.97 (**C2**), 33.93 (**C8**), 65.31 (**C1**), 115.25 (**C10**), 116.48 (**C1′**), 117.16 (**C3′**), 122.12 (**C8′**), 128.17 (**C7′**), 129.18 (**C4′**), 139.13 (**C9**), 144.95 (**C1′**), 147.23 (**C5′**, **C6′**), 167.13 (**C=O**). Analysis (%): calculated for C_19_H_26_O_4_: C 71.67, H 8.23. Found: C 71.91, H 8.66.

Hex-5′-enyl-3-(3,5-dimethoxy-4-hydroxyphenyl)prop-2-enoic (**9b**)

The general procedure for the preparation of the monocatenar ester with 5-hexen-1-ol (1 mL; 0.74 mmol; 1 eq.) and sinapic acid (1 g; 4.44 mmol; 6 eq.) in 30 mL of acetone is as follows. Compound **9b** was obtained as an orange-yellow oil with a yield of 40%.

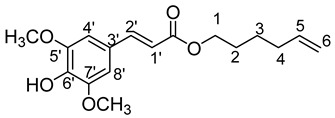



ν_max_ (ATR) cm^−1^: 3392 (OH), 2934 (C–H), 1732 (C=O), 1612 (–CH=CH_2_–), 1665 (Phenyl-CH=CH-COO). ^1^H NMR (500 MHz; CD_3_OD, ppm) δ 0.58 (2H, m, **H3**), 1.62 (2H, m, **H2**), 2.19 (2H, m, **H4**), 3.83 (6H, t, J 7.2 Hz, (**CH_3_-O**)**_2_**-Phenyl), 3.99 (2H, d, J 7.2 Hz, **H1**), 5.12 (1 H, d, J 7.2 Hz, **H6**), 5.82 (1H, m, **H1′**), 6.32 (1H, d, J 7.2 Hz, **H5**), 6.72 (2H, s, **H4′**, **H8′**), 7.48 (1H, d, J 7.2 Hz, **H2′**). ^13^C NMR (125 MHz; DMSO-d_6_, ppm) δ 25.2 (**C3**), 25.6 (**C2**), 33.7 (**C4**), 56.3 ((**CH_3_-O**)**_2_**-Phenyl), 65.1 (**C1**), 108.2 (**C6**), 115.2 (**C1′**), 116.1 (**C3′**), 126.1 (**C4′**, **C8′**), 136.0 (**C6′**),139.2 (**C5**), 146.3 (**C2′**), 148.1 (**C5′**, **C7′**), 167.9 (**C=O**). Analysis (%): calculated for C_17_H_22_O_5_: C 66.65, H 7.24. Found: C 66.39, H 7.62.

Hex-5′-enyl-3-(4-hydroxy-3-méthoxyphényl)prop-2-enoïc (**10b**)

The general procedure for the preparation of the monocatenar ester with 5-hexen-1-ol (0.7 mL; 6.53 mmol; 1 eq.) and ferulic acid or 3-(4-hydroxy-3-methoxyphenyl)prop-2-enoic (7.61 mg; 39.2 mmol; 6 eq.) in 60 mL of 2-methyl-2-butanol is as follows. Compound **10b** was obtained as a clear oil with a yield of 52%.

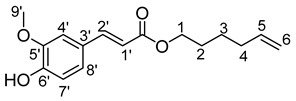



ν_max_ (ATR) cm^−1^: 3392 (OH), 2927 (C–H), 1713 (C=O), 1659–1640 (CH=CH_2_), 1688–1664 (Phenyl-**CH=CH**-COO). ^1^H NMR (500 MHz; CD_3_OD, ppm) δ 1.48 (2H, m, **H3**), 1.67 (2H, m, **H4**), 2.09 (2H, m, **H2**), 3.82 (3H, s, **CH_3_**-O-Phenyl), 4.14 (2H, t, J 7.2 Hz, **H1**), 5.05 (2H, m, **H6**), 5.86 (1H, m, **H5**), 6.49 (1H, d, J 7.2 Hz, **H7′**), 6.80 (1H, d, J 7.2 Hz, **H1′**), 7.13 (1H, d, J 7.2 Hz, **H2′**), 7.33 (1H, s, **H4′**), 7.56 (1H, d, J 7.2, **H8′**). ^13^C NMR (125 MHz; DMSO-d_6_, ppm) δ 25.2 (**C3**), 28.3 (**C4**), 33.2 (**C2**), 56.2 (**CH_3_**-O-Phenyl), 64.0 (**C1**), 111.6 (**C7′**), 114.9 (**C1′**), 115.5 (**C6**), 115.9 (**C4′**), 123.6 (**C2′**), 126.0 (**C3′**), 138.9 (**C5**), 145.4 (**C8′**), 149.8 (**C6′**), 148.4 (**C5′**), 167.2 (**C=O**). Analysis (%): calculated for C_16_H_20_O_4_: C 69.55, H 7.30. Found: C 69.73, H 7.52.

Dec-9′-enyl-3-enyl-3-(4-hydroxy-3-méthoxyphényl)prop-2-enoïc (**11b**)

The general procedure for the preparation of the monocatenar ester with 9-decen-1-ol (1.2 mL; 3.01 mmol; 1 eq.) and 3-(4-hydroxy-3-methoxyphenyl)prop-2-enoic acid (3.51 mg; 18.06 mmol; 6 eq.) in 60 mL of 2-methyl-2-butanol is as follows. Compound **11b** was obtained as a clear oil with a yield of 52%.

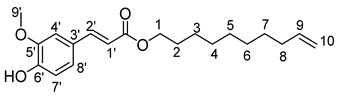



ν_max_ (ATR) cm^−1^: 3394 (OH), 2926 (C–H), 1724 (C=O), 1660–1640 (CH=CH_2_), 1690–1664 (Phenyl-**CH=CH**-COO). ^1^H NMR (500 MHz; CD_3_OD, ppm) δ 1.33 (8H, m, **H4**, **H5**, **H6**, **H7**), 1.55 (2H, m, **H3**), 1.63 (2H, m, **H8**), 2.02 (2H, m, **H2**), 3.82 (3H, s, **CH_3_**-O-Phenyl), 4.12 (2H, t, J 7.2 Hz, **H1**), 4.99 (2H, m, **H10**), 5.82 (1H, m, **H9**), 6.47 (1H, d, J 7.2 Hz, **H7′**), 6.80 (1H, d, J 7.2 Hz, **H1′**), 7.11 (1H, d, J 7.2 Hz, **H2′**), 7.31 (1H, s, **H4′**), 7.56 (1H, d, J 7.2, **H8′**). ^13^C NMR (125 MHz; DMSO-d_6_, ppm) δ 28.9 (**C5**), 28.9 (**C6**), 29.0 (**C4**), 29.1 (**C7**), 29.2 (**C3**), 29.3 (**C8**), 33.6 (**C2**), 56.1 (**CH_3_**-O-Phenyl), 64.2 (**C1**), 111.5 (**C7′**), 114.9 (**C1′**), 115.0 (**C6**), 115.9 (**C4′**), 123.5 (**C2′**), 126.0 (**C3′**), 139.2 (**C5**), 145.4 (**C8′**), 148.4 (**C6′**), 149.8 (**C5′**), 167.1 (**C=O**). Analysis (%): calculated for C_20_H_28_O_4_: C 72.26, H 8.49. Found: C 71.92, H 8.64.

### 3.7. General Procedure for the Preparation of the Fatty Ester-Based Bolaamphiphiles under Microwave Activation

In a microwave tube, the fatty ester in CH_2_Cl_2_ (10 mL) was dissolved under argon and the Grubbs II catalyst was then added in three portions over the whole reaction time. The mixture was irradiated for 40 min at a power of 60 W and a temperature of 60 °C. After 40 min of reaction, the reaction medium was treated with activated charcoal to remove the Grubbs catalyst and filtered through celite. After evaporation of the solvent under reduced pressure, the residue was purified by a flash chromatography system coupled with a UV detector with elution mixture CH_2_Cl_2_/MeOH (9/1) over 35 min.

1′,10′-bis-dec-5′-enyl-3-(4-hydroxyphenyl)propionic ester (**12b**)

The general procedure for the preparation of the fatty ester-based bolaamphiphiles with compound **1b** (1 g, 4.03 mmol, 1 eq.) under microwave activation with Grubbs II catalyst (0.34 g, 0.403 mmol; 0.1 eq.) dissolved in 10 mL CH_2_Cl_2_ is as follows. Compound **12b** is obtained as a brown paste with a yield of 56%.

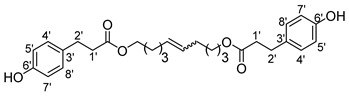



ν_max_ (ATR) cm^−1^: 3385 (OH), 2927–2855 (C–H), 1730 (C=O), 1614 (C=C). ^1^H NMR (500 MHz; CD_3_OD, ppm) δ 1.27–1.36 (12H, m, **H3**, **H4**, H**2**), 2.51 (4H, t, J 7.2 Hz, H**2′**), 2.74 (4H, t, J 7.2 Hz, H**1′**), 3.98 (4H, t, J 7.2 Hz, H**1**), 5.74 (2H, d, J 7.2 Hz, H**5**), 6.71 (4H, d, J 10 Hz, H**4′**, H**8′**), 6.96 (4H, d, J 10 Hz, H**5′**, H**7′**). ^13^C NMR (125 MHz; DMSO-d_6_, ppm) δ 25.9 (2 **C3**), 28.2 (2 **C4**), 30.1 (2 **C2**), 33.4 (2 **C2′**), 36.4 (2 **C1′**), 64.2 (2 **C1**),116.2 (2 **C4′**, 2 **C8′**), 129.5 (2 **C5′**, 2 **C7′**), 130.1 (2 **C3′**), 130.5 (CH_2_**CH**(**Z**)**=CH**(**Z**)CH_2_), 130.9 (CH_2_**CH**(**E**)**=CH**(**E**)CH_2_), 156.1 (2 **C6′**), 172.8 (**C=O**). Analysis (%): calculated for: C_28_H_36_O_6_: C 71.77, H 7.74. Found: C 72.04, H 7.44%.

1′,12′-bis-dodec-6′-enyl-3-(4-hydroxyphenyl)propionic ester (**13b**)

The general procedure for the preparation of the fatty ester-based bolaamphiphiles with compound **2b** (0.45 g, 1.72 mmol, 1 eq.) under microwave activation with Grubbs II catalyst (0.15 g, 0.172 mmol; 0.1 eq.) dissolved in 10 mL CH_2_Cl_2_ is as follows. Compound **13b** is obtained as a brown paste with a yield of 48%.

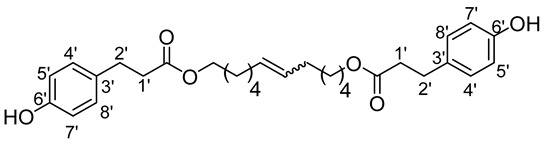



ν_max_ (ATR) cm^−1^: 3374 (OH), 2923–2835 (C–H), 1732 (C=O), 1621 (C=C). ^1^H NMR (500 MHz; CD_3_OD, ppm) δ 1.28–1.32 (12H, m, **H3**, **H4**, **H5**, **H2**), 2.53 (4H, t, J 7.2 Hz, **H2′**), 2.72 (4H, t, J 7.2 Hz, **H1′**), 4.08 (4H, t, J 7.2 Hz, **H1**), 5.84 (2H, d, J 7.2 Hz, **H6**), 6.73 (4H, d, J 10 Hz, **H4′**, **H8′**), 6.92 (4H, d, J 10 Hz, **H5′**, **H7′**). ^13^C NMR (125 MHz; DMSO-d_6_, ppm) δ 25.9 (2**C3**), 28.3 (2 **C4**), 28.6 (2 **C5**), 32.2 (2 **C2**), 33.58 (2 **C2′**), 36.04 (2 **C1′**), 64.25 (2 **C1**),115.18 (2 **C4′**, 2 **C8′**), 129.4 (2 **C5′**, 2 **C7′**), 130.2 (2**C3′**), 130.5 (CH_2_**CH**(**Z**)**=CH**(**Z**)CH_2_), 130.9 (CH_2_**CH**(**E**)**=CH**(**E**)CH_2_), 156.2 (2 **C6′**), 173.0 (**C=O**). Analysis (%): calculated for C_30_H_40_O_6_: C 72.55, H 8.12. Found: C 72.06, H 7.93.

1′,18′-bis-octadec-9′-enyl-3-(4-hydroxyphenyl)propionic ester (**14b**)

The general procedure for the preparation of the fatty ester-based bolaamphiphiles with compound **3b** (1 g, 3.29 mmol, 1 eq.) under microwave activation with Grubbs II catalyst (0.28 g, 0.329 mmol; 0.1 eq.) dissolved in 10 mL CH_2_Cl_2_ is as follows. Compound **14b** is obtained as a brown paste with a yield of 60%.

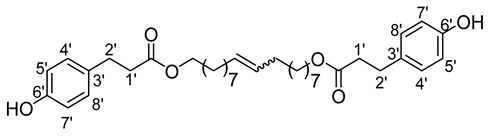



ν_max_ (ATR) cm^−1^: 3383 (OH), 2927–2854 (C–H), 1731 (C=O), 1614 (C=C). ^1^H NMR (500 MHz; CD_3_OD, ppm) δ 1.29 (20H, m, **H3**, **H4**, **H5**, **H6**, **H7**), 1.51 (4H, m, J 7.2 Hz, **H2**), 1.94 (4H, t, J 7.2 Hz, **H8**), 2.53 (4H, t, J 7.2 Hz, **H1′**), 2.72 (4H, t, J 7.2 Hz, **H2′**), 3.97 (4H, t, J 10 Hz, **H1**), 5.36 (2H, t, J 7.2 Hz, **H9**), 6.65 (4H, d, J 10 Hz, **H5′**, **H7′**), 6.90 (4H, d, J 10 Hz, **H4′**, **H8′**). ^13^C NMR (125 MHz; DMSO-d_6_, ppm) δ 28.9 (2 **C5**), 29.1 (2 **C6**), 29.2 (2 **C4**), 29.4 (2 **C7**), 29.4 (2 **C3**), 30.0 (2 **C8**), 32.4 (2 **C2**), 32.4 (2 **C2′**), 36.0 (2 **C1′**), 64.2 (2 **C1**), 115.5 (2 **C4′**, 2 **C8′**), 129.5 (2 **C5′**, 2 **C7′**), 130.1 (2 **C3′**), 130.5 (CH_2_**CH**(**Z**)**=CH**(**Z**)CH_2_), 130.9 (CH_2_**CH**(**E**)**=CH**(**E**)CH_2_), 156.1 (**C6′**), 172.8 (**C=O**). Analysis (%): calculated for C_36_H_52_O_6_: C 74.45, H 9.02. Found: C 74.07, H 8.78.

1′,10′-bis-dec-5′-enyl-3-(4-hydroxyphenyl)prop-2-enoic (**15b**)

The general procedure for the preparation of the fatty ester-based bolaamphiphiles with compound **4b** (0.1 g, 4.06 mmol, 1 eq.) under microwave activation with Grubbs II catalyst (0.35 g, 0.406 mmol; 0.1 eq.) dissolved in 10 mL CH_2_Cl_2_ is as follows. Compound **14b** is obtained as a brown paste with a yield of 42%.

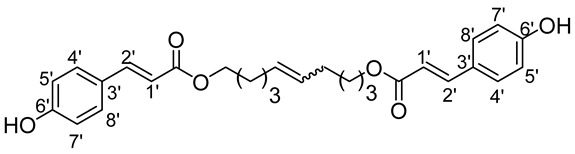



ν_max_ (ATR) cm^−1^: 3395 (OH), 2937 (C–H), 1731 (C=O), 1614 (C=C), 1668 (Phenyl-**CH=CH**-COO). ^1^H NMR (500 MHz; CD_3_OD, ppm) δ 1.25–1.51 (12H, m, **H3**, **H4**, **H2**), 4.25 (4H, t, J 7.2 Hz, **H1**), 5.48 (2H, t, J 7.2 Hz, **H5**), 6.30 (2H, d, J 7.2 Hz, **H2′**), 6.81 (4H, d, J 10 Hz, **H5′**, **H7′**), 7.30 (2H, d, J 7.2 Hz, **H1′**), 7.74 (4, d, 10 Hz, **H4′**, **H8′**). ^13^C NMR (125 MHz; DMSO-d_6_, ppm) δ 24.1 (2 **C3**), 25.1 (2 **C4**), 31.3 (2 **C2**), 32.2 (2 **C2′**), 65.3 (2 **C1**), 114.1 (2 **C2′**), 1115.0 (2 **C5′**, 2 **C7′**), 127.2 (2 **C3′**), 129.6 (2 **C4′**, 2 **C8′**), 130.1 (CH_2_**CH**(**Z**)**=CH**(**Z**)CH_2_), 130.3 (CH_2_**CH**(**E**)**=CH**(**E**)CH_2_), 145.4 (2 **C6′**), 158.2 (2 **C1′**), 170.5 (**C=O**). Analysis (%): calculated for C_28_H_32_O_6_: C 72.39, H 6.94. Found: C 72.82, H 7.21.

### 3.8. General Procedure for the Preparation of the Unsymmetrical Fatty Ester- and Rhamnoside-Based Bolaamphiphiles by Classic Heating

The rhamnoside-based bolaamphiphile and the fatty ester were dissolved in CH_2_Cl_2_ (35 mL)/MeOH (5 mL) in a Schlenk tube under argon and the Grubbs II catalyst was added in three portions over the whole reaction time. The mixture was irradiated at a temperature of 45 °C. After 24 h of reaction, the reaction medium was treated with activated charcoal to remove the Grubbs catalyst and filtered through celite. After evaporation, the residue was purified by a flash chromatography system coupled with a UV detector with elution mixture CH_2_Cl_2_/MeOH (9/1) over 45 min.

1′,10′-bis-dec-5′-enyl-3-(4-hydroxyphenyl)propionicacid–α–L-rhamnopyranoside (**1ab**)

The general procedure for the preparation of the unsymmetrical fatty ester- and rhamnoside-based bolaamphiphiles with compound **6a** (1 g, 2.15 mmol, 2 eq.) and compound **1b** (0.267 g, 1.080 mmol) under argon with Grubbs II catalyst (0.09 g, 0.108 mmol; 0.05 eq.) dissolved in 40 mL CH_2_Cl_2_/MeOH (35/5) is as follows. Compound **1ab** is obtained as a brown paste with a yield of 65%.

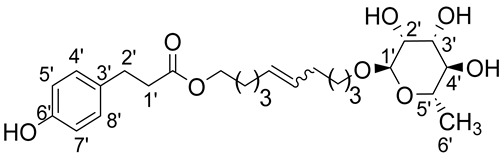



ν_max_ (ATR) cm^−1^: 3353 (OH), 2928 (C–H), 1732 (C=O), 1615 (C=C). [α]^20^_D_ (589 nm, MeOH) = −26.601. ^1^*H NMR* (500 MHz; *DMSO-d*_6_, ppm) δ 1.27 (3H, d, *J* 7.5 Hz, **H6′_-ose_**), 1.41 (4H, m, **H3**), 1.83 (4H, m, **H2**), 2.15 (4H, m, **H4**), 2.53 (2H, t, *J* 7.2 Hz, **H2′_-acid_**), 2.72 (2H, t, *J* 7.2 Hz, **H1′_-acid_**), 3.12–3.54 (5H, m, **H1′_-ose_**, **C2′_-ose_**, **C3′_-ose_**, **C4′_-ose_**, **C5′_-ose_**), 4.01 (2H, t, *J* 7.2 Hz, **H1_-acid_**), 4.52–4.72 (3H, s, 3 **OH_-ose_**), 5.76 (2H, t, *J* 7.2 Hz, 2 **H5_-C=C-_**)_,_ 6.71 (2H, d, *J* 10 Hz, **H4′**, **H8′**), 6.98 (2H, d, *J* 10 Hz, **H5′**, **H7′**). ^13^*C NMR* (125 MHz; *DMSO-d*_6_, ppm) δ 17.7 (**C6′_-ose_**), 25.8 (2 **C3**), 28.6, 31.7 (2 **C2**, 2 **C4**), 32.9 (**C2′_-acid_**), 36.1 (**C1′_-acid_**), 63.9 (**C1_-acid_**), 66.3 (**C1_-ose_**), 68.7–74.1 (**C2′_-ose_**, **C3′_-ose_**, **C4′_-ose_**, **C5′_-ose_**), 100.4 (**C1′_-ose_**), 115.4 (**C4′_-acid_**, **C8′_-acid_**), 115.5 (**C5′_-acid_**, **C7′_-acid_**), 131.0 (CH_2_**CH**(**Z**)**=CH**(**Z**)CH_2_), 131.5 (CH_2_**CH**(**E**)**=CH**(**E**)CH_2_), 136.3 (**C3′**), 156.1 (**C6′**), 172.8 (**C=O**). Analysis (%): calculated for C_25_H_38_O_8_: C 64.36, H 8.21. Found: C 64.09, H 8.57.

1′,12′-bis-dodec-6′-enyl-3-(4-hydroxyphenyl)propionicacid–α–l-rhamnopyranoside (**2ab**)

The general procedure for the preparation of the unsymmetrical fatty ester- and rhamnoside-based bolaamphiphiles with compound **6a** ((0,20 g, 0,4 mmol, 2 éq.)) and compound **2b** (0.053 g, 0.2 mmol, 1 eq.) under argon with Grubbs II catalyst (0.017 g, 0.02 mmol; 0.05 eq.) dissolved in 40 mL CH_2_Cl_2_/MeOH (35/5) is as follows. Compound **1ab** is obtained as a brown paste with a yield of 42%.

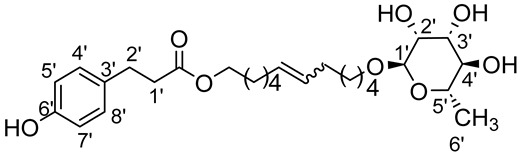



ν_max_ (ATR) cm^−1^: 3351 (OH), 2923 (C–H), 1731 (C=O), 1614 (C=C). [α${]}_{D}^{20}$ (589 nm, MeOH) = −16.900. ^1^*H NMR* (500 MHz; DMSO-d_6_, ppm) δ 1.16 (3H, d, J 7.5 Hz, **H6′_-ose_**), 1.28 (4H, m, **H4**) 1.43 (4H, m, **H3**), 1.80 (4H, m, **H2**), 2.12 (4H, m, **H4**), 2.56 (2H, t, J 7.2 Hz, **H2′_-acid_**), 2.70 (2H, t, J 7.2 Hz, **H1′_-acid_**), 3.14–3.52 (5H, m, **H1_-ose_**, **H2′_-ose_**, **H3′_-ose_**, **H4′_-ose_**, **H5′_-ose_**), 4.13 (2H, t, J 7.2 Hz, **H1_-acid_**), 4.42–4.82 (3H, s, 3 **OH_-ose_**), 5.74 (2H, t, J 7.2 Hz, **H5_-C=C-_**)_,_ 6.67 (2H, d, J 10 Hz, **H4′**, **H8′**), 7.08 (2H, d, J 10 Hz, **H5′**, **H7′**). ^13^C NMR (125 MHz; DMSO-d_6_, ppm) δ 17.3 (**C6′_-ose_**), 25.4 (2 **C4**), 25.7 (2 **C3**), 28.6, 31.7 (2 **C2**, 2 **C4**), 32.8 (**C2′_-acid_**), 36.2 (**C1′_-acid_**), 63.9 (**C1_-acid_**), 66.1 (**C1_-ose_**), 68.5–74.1 (**C2′_-ose_**, **C3′_-ose_**, **C4′_-ose_**, **C5′_-ose_**), 100.6 (**C1′_-ose_**), 115.1 (**C4′_-acid_**, **C8′_-acid_**), 114.3 (**C5′_-acid_**, **C7′_-acid_**), 130.9 (CH_2_**CH**(**Z**)**=CH**(**Z**)CH_2_), 131.6 (CH_2_**CH**(**E**)**=CH**(**E**)CH_2_), 136.3 (**C3′**), 156.4 (**C6′**), 172.8 (**C=O**). Analysis (%): calculated for C_27_H_42_O_8_: C 65.56, H 8.56. Found: C 65.80, H 8.74.

1′,18′-bis-dodec-9′-enyl-3-(4-hydroxyphenyl)propionicacid–α–l-rhamnopyranoside (**3ab**)

The general procedure for the preparation of the unsymmetrical fatty ester- and rhamnoside-based bolaamphiphiles with compound **8a** (1 g, 1.73 mmol, 2 eq.) and compound **3b** (0.264 g, 0.867 mmol) under argon with Grubbs II catalyst (0.074 g, 0.086 mmol; 0.05 eq.) dissolved in 40 mL CH_2_Cl_2_/MeOH (35/5) is as follows. Compound **3ab** is obtained as a brown paste with a yield of 68%.

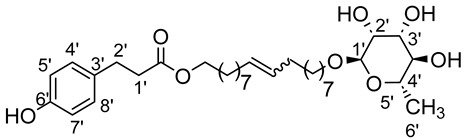



ν_max_ (ATR) cm^−1^: 3353 (OH), 2925 (C–H), 1732 (C=O), 1614 (C=C). [α${]}_{D}^{20}$ (589 nm, MeOH) = −32.601. ^1^H NMR (500 MHz; DMSO-d_6_, ppm) δ 1.12 (3H, d, J 7.5 Hz, **H6′_-ose_**), 1.34 (20H, m, **H3**, **H4**, **H5**, **H6**, **H7**), 1.52 (4H, m, **H2**), 2.03 (4H, m, **H8**), 2.51 (2H, t, J 7.2 Hz, **H2′_-acid_**), 2.74 (2H, t, J 7.2 Hz, **H1′_-acid_**), 3.18–3.47 (5H, m, **H1_-ose_**, **H2′_-ose_**, **H3′_-ose_**, **H4′_-ose_**, **H5′_-ose_**), 4.06 (2H, t, J 7.2 Hz, **H1_-acide_**), 4.13–4.51 (3H, s, 3 **OH_-ose_**), 5.81 (2H, t, J 7.2 Hz, **H9_-C=C-_**)_,_ 6.72 (2H, d, J 10 Hz, **H4′**, **H8′**), 7.02 (2H, d, J 10 Hz, **H5′**, **H7′**). ^13^C NMR (125 MHz; DMSO-d_6_, ppm) δ 18.3 (**C6′_-ose_**), 26.1, 28.7, 28.9, 29.2, 29.4 (2 **C3**, 2 **C4**, 2 **C5**, 2 **C6**, 2 **C7**), 29.4 (2 **C2**), 31.8 (2 **C8**), 33.4 (**C2′_-acid_**), 36.2 (**C1′_-acid_**), 64.2 (**C1_-acid_**), 66.8 (**C1_-ose_**), 68.8–73.5 (**C2′_-ose_**, **C3′_-ose_**, **C4′_-ose_**, **C5′_-ose_**), 100.7 (**C1′_-ose_**), 115.5 (**C4′_-acid_**, **C8′_-acid_**), 128.3 (**C5′_-acid_**, **C7′_-acid_**), 130.7 (CH_2_**CH**(**Z**)**=CH**(**Z**)CH_2_), 131.1 (CH_2_**CH**(**E**)**=CH**(**E**)CH_2_), 138.5 (**C3′_-acid_**),156.2 (**C6′**), 171.8 (**C=O**). Analysis (%): calculated for: C_33_ H_54_O_8_: C 68.48, H 9.40. Found: C 67.98, H 9.78.

1′,20′-bis-eicosa-10′-enyl-3-(4-hydroxyphenyl)propionicacid–α–l-rhamnopyranoside (**4ab**)

The general procedure for the preparation of the unsymmetrical fatty ester- and rhamnoside-based bolaamphiphiles with compound **10a** (0.5 g, 0.872 mmol, 2 eq.) and compound **4b** (0.132 g, 0.437 mmol, 1 eq.) under argon with Grubbs II catalyst (0.017 g, 0.019 mmol; 0.05 eq.) dissolved in 40 mL CH_2_Cl_2_/MeOH (35/5) is as follows. 

Compound **4ab** is obtained as a brown paste with a yield of 46%.

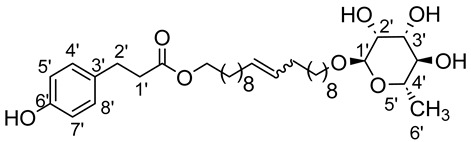



ν_max_ (ATR) cm^−1^: 3350 (OH), 2921 (C–H), 1729 (C=O), 1612 (C=C). [α${]}_{D}^{20}$ (589 nm, MeOH) = −20,300. ^1^H NMR (500 MHz; DMSO-d_6_, ppm) δ 1.15 (3H, d, J 7.5 Hz, **H6′_-ose_**), 1.32 (24H, m, **H3**, **H4**, **H5**, **H6**, **H7**, **H8**), 1.50 (4H, m, **H2**), 2.31 (4H, m, **H9**), 2.58 (2H, t, J 7.2 Hz, **H2′_-acid_**), 2.73 (2H, t, J 7.2 Hz, **H1′_-acid_**), 3.14–3.52 (5H, m, **H1_-ose_**, **H2′_-ose_**, **H3′_-ose_**, **H4′_-ose_**, **H5′_-ose_**), 4.09 (2H, t, J 7.2 Hz, **H1_-acide_**), 4.15–4.73 (3H, s, 3 **OH_-ose_**), 5.95 (2H, t, J 7.2 Hz, **H10_-C=C-_**)_,_ 6.74 (2H, d, J 10 Hz, **H4′**, **H8′**), 7.08 (2H, d, J 10 Hz, **H5′**, **H7′**). ^13^C NMR (125 MHz; DMSO-d_6_, ppm) δ 17.5 (**C6′_-ose_**), 24.8, 27.6, 28.1, 28.9, 29.2, 29.3 (2 **C3**, 2 **C4**, 2 **C5**, 2 **C6**, 2 **C7**, 2 **C8**), 29.7 (2 **C2**), 32.1 (2 **C9**), 34.8 (**C2′_-acid_**), 36.1 (**C1′_-acid_**), 64.5 (**C1_-acid_**), 67.0 (**C1_-ose_**), 68.1–74.0 (**C2′_-ose_**, **C3′_-ose_**, **C4′_-ose_**, **C5′_-ose_**), 105.7 (**C1′_-ose_**), 115.0 (**C4′_-acid_**, **C8′_-acid_**), 129.2 (**C5′_-acid_**, **C7′_-acid_**), 130.6 (CH_2_**CH**(**Z**)**=CH**(**Z**)CH_2_), 131.1 (CH_2_**CH**(**E**)**=CH**(**E**)CH_2_), 137.7 (**C3′_-acid_**),154.5 (**C6′**), 171.8 (**C=O**). Analysis (%): calculated for: C_35_ H_58_O_8_: C 69.27, H 9.63. Found: C 69.38, H 9.71.

## 4. Conclusions

In this study, we developed greener syntheses of six monocatenar and five bolaform rhamnosides, respecting the fundamental principles of green chemistry, which, when coupled with a CombiFlash purification technique, allowed the obtention of compounds with good to very good yields. We have also prepared 10 phenolic acid esters by an esterification reaction, as well as four corresponding bolaforms via a cross-metathesis reaction with good yields. Four dissymmetric bolaforms derived from phloretic esters and rhamnosides have been also easily prepared.

Two monocatenar rhamnosides, as well as a dissymmetrical bolaform (phloretic ester/rhamnoside) trigger a plant defense response in Arabidopsis. Phenolic acid esters have shown good antioxidant activities and we observed that the levels of cytotoxicity depend on the carbon chains we used. The transformation into corresponding bolaforms or into dissymmetric bolaforms associated with rhamnosides significantly decreases their toxicity while retaining their antioxidant properties. These aspects can therefore augur a potential use of some of these compounds in the field of cosmetics. An in-depth study of the mechanisms involved in planta and protection assays will, however, be needed prior to determining the potential use of these compounds in plant protection and pest management strategies. Biophysical studies on their interaction with plant and skin biomimetic plasma membranes would also help to better understand their mode of action at the cell level. An in vitro study on dermal cells proved that L-rhamnose derivatives are less cytotoxic than those from phloretic acid, and that the carbon chain length plays an important role. Moreover, bolaform structures issued from rhamnosides and phloretic esters are less toxic. Further tests on other cell models will be carried out in order to use these compounds in different fields of application.

## Figures and Tables

**Table 1:** Preliminary results for L-rhamnose glycosylation under classic thermic conditions.

Entry	Alcohol	Conditions	Rhamnoside/Isolated Yields (%) after Flash Chromatography (CH_2_Cl_2_/MeOH: 9/1)
1	Hex-5-enol	THF, 80 °C, 48 h	**1a**/19
2	Dec-9-enol	THF, 80 °C, 48 h	**5a**/10
3	Hex-5-enol	THF, 60 °C, 48 h	**1a**/5
4	Dec-9-enol	THF, 60 °C, 48 h	**5a**/5
5	Hex-5-enol	THF, 60 °C, 48 h Addition of the catalyst 3 times	**1a**/7
6	Dec-9-enol	THF, 60 °C, 48 h Addition of the catalyst 3 times	**5a**/7
7	Hex-5-enol	Neat, 60 °C, 5 h	**1a**/38
8	Dec-9-enol	Neat, 60 °C, 5 h	**5a**/40

L-rhamnose (1 eq.), alcohol (2 eq.), PTSA (0.6 eq.).

**Table 2:** Glycosylation of L-rhamnose with hex-5-enol in THF (5 mL) under microwave conditions.

Entry	Ratios (eq.) L-Rhamnose/Hex-5-enol/PTSA	Conditions	Isolated Yields in 1a (%) (Combiflash)
1	1/2/0.6	35 °C, 5 min, 80 W	-
2	1/2/0.6	35 °C, 10 min, 80 W	17
3	1/2/0.6	80 °C, 35 min, 60 W	40
4	1/2/0.6	60 °C, 60 min, 60 W	48
5	1/2/0.6	80 °C, 60 min, 60 W	50
6	1/4/0.6	60 °C, 60 min, 60 W	58
7	1/4/0.6	80 °C, 60 min, 60 W	55
8	1/4/0.6	60 °C, 120 min, 60 W	64
9	1/6/0.6	60 °C, 120 min, 60 W	62
10	1/6/0.6	80 °C, 60 min, 60 W	60

**Table 3:** Glycosylation of L-rhamnose under microwaves (60 °C, 120 min, 60 W).

Entry	Alcohol	Solvent (5 mL)	Rhamnoside	Isolated Yields (%) (Combiflash)
1	Hex-5-enol	THF	**1a**	64
2	Hex-5-enol	γ-valerolactone	**1a**	66
3	Hex-5-enol	2-methyltetrahydrofuran	**1a**	63
4	Hept-6-enol	THF	**2a**	60
5	Oct-7-enol	THF	**3a**	50
6	Non-8-enol	THF	**4a**	53
7	Dec-9-enol	THF	**5a**	48
8	Dec-9-enol	γ-valerolactone	**5a**	73
9	Dec-9-enol	2-methyltetrahydrofuran	**5a**	55
10	Undec-10-enol	THF	**6a**	47

**Table 4:** Cross metathesis of rhamnosides.

Entry	Monocatenar Rhamnoside	Conditions	Grubbs Catalyst (0.1 eq.)	Purification	Bolaform Rhamnoside Isolated Yields (%)
1	**1a**	CH_2_Cl_2_	Grubbs I	Silica Chromatography	**7a** 19
2	**2a**	CH_2_Cl_2_	Grubbs I	Silica Chromatography	**8a** 13
3	**5a**	CH_2_Cl_2_	Grubbs I	Silica Chromatography	9a 10
4	**1a**	CH_2_Cl_2_/MeOH (9/1)	Grubbs I	CombiFlash	**7a** 52
5	**2a**	CH_2_Cl_2_/MeOH (9/1)	Grubbs I	CombiFlash	**8a** 37
6	**5a**	CH_2_Cl_2_/MeOH (9/1)	Grubbs I	CombiFlash	**9a** 31
7	**1a**	CH_2_Cl_2_/MeOH (9/1)	Grubbs II	CombiFlash	**7a** 77
8	**2a**	CH_2_Cl_2_/MeOH (9/1)	Grubbs II	CombiFlash	**8a** 60
9	**5a**	CH_2_Cl_2_/MeOH (9/1)	Grubbs II	CombiFlash	**9a** 56
10	**6a**	CH_2_Cl_2_/MeOH (9/1)	Grubbs II	CombiFlash	**10a** 32

**Table 5:** Enzymatic catalyzed esterification of phloretic acids.

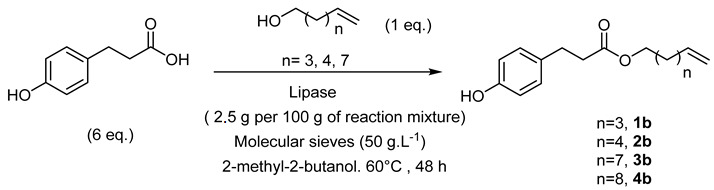			
Compounds	Conversion of Acid (% GC)	Isolated Yields (Combiflash) %	Isolated Yields (Flash Chromatography) %
**1b**	97	95	12
**2b**	90	88	28
**3b**	97	96	46
**4b**	85	79	31

**Table 6:** Esterification of paracoumaric, ferulic, caffeic and sinapic acids (conditions: acid (6 eq.) and alcohol (1 eq., lipase 2.5 g per 100 g of reaction mixture, molecular sieves (50 g·L^−1^), 60 °C, 48 h).

Starting Phenolic Acid	Compounds	Solvent	Isolated Yields (%)
*p*-coumaric acid	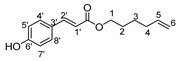 **5b**	2M2B (40 mL)	70
*p*-coumaric acid	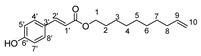 **6b**	2M2B (40 mL)	74
caffeic acid	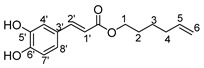 **7b**	2M2B/THF (1/2) (30 mL)	52
caffeic acid	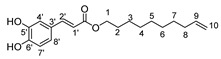 **8b**	2M2B/THF (1/2) (30 mL)	45
ferulic acid	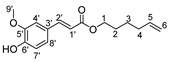 **10b**	2M2B (60 mL)	52
ferulic acid	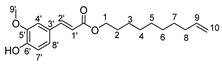 **11b**	2M2B (60 mL)	52
sinapic acid	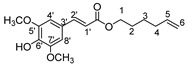 **9b**	Acetone (30 mL)	40

**Table 7:** Metathesis of phenolic esters.

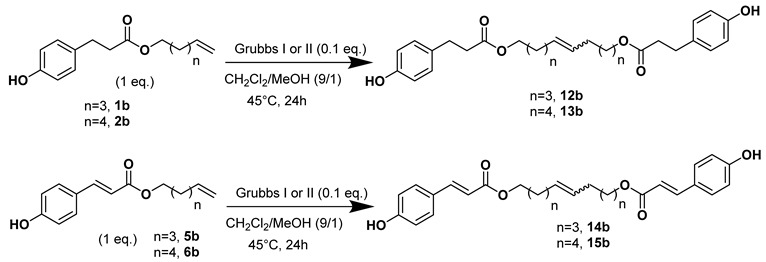		
Compound	Isolated Yields	
	Grubbs I Catalyst	Grubbs II Catalyst
**12b**	20	56
**13b**	16	48
**14b**	18	60
**15b**	11	42

**Table 8:** Antioxidant properties of phenolic (di)esters.

Entry	Compounds	IC50 (μmol/L)
1	Vitamin C	34
2	**5b**	45
3	**7b**	29
4	**9b**	31
5	**10b**	47
6	**6b**	32
7	**11b**	32
8	**12b**	36
9	**14b**	35
10	**15b**	27

## Data Availability

Not applicable.

## Sample Availability

Samples of all synthesized compounds are available from the authors.
